# Temporal Interactome Mapping of Human Tau in Drosophila Reveals Progressive Mitochondrial Engagement and Porin/VDAC1-Dependent Modulation of Toxicity

**DOI:** 10.3390/ijms26199741

**Published:** 2025-10-07

**Authors:** Eleni Tsakiri, Martina Samiotaki, Efthimios M. C. Skoulakis, Katerina Papanikolopoulou

**Affiliations:** 1 Institute for Fundamental Biomedical Research, Biomedical Sciences Research Centre “Alexander Fleming”, 16672 Athens, Greece; etsakiri@med.uoa.gr (E.T.); skoulakis@fleming.gr (E.M.C.S.); 2 Institute for Bio-Innovation, Biomedical Sciences Research Centre “Alexander Fleming”, 16672 Vari, Greece; samiotaki@fleming.gr

**Keywords:** Tau protein, Drosophila, proteomics, mitochondria, porin

## Abstract

Tau protein misfolding and aggregation are central to Tauopathies, yet the temporal dynamics of Tau interactions in vivo remain poorly understood. Here, we applied quantitative proteomics to demonstrate that the interactome of human Tau in adult Drosophila brains changes dynamically over a 12-day time course, revealing a progressive shift from early cytosolic and ribosomal associations to late enrichment of mitochondrial and synaptic partners. Notably, the mitochondrial pore protein Porin/VDAC1 was identified as a late-stage interactor and functional analyses demonstrated that Tau overexpression impairs mitochondrial respiration, elevates oxidative damage, and disrupts carbohydrate homeostasis. To validate this temporally specific interaction, Porin was downregulated, resulting in reduced Tau mitochondrial association, phosphorylation and aggregation. Paradoxically, however, Porin attenuation exacerbated Tau-induced toxicity, including shortened lifespan, locomotor deficits, and impaired learning. These findings indicate that while Porin facilitates pathological Tau modifications, it is also essential for neuronal resilience, highlighting a complex role in modulating Tau toxicity. Our study provides a temporal map of Tau-associated proteome changes in vivo and identifies mitochondria as critical mediators of Tau-driven neurodegeneration.

## 1. Introduction

Tau is a soluble, intrinsically disordered microtubule-associated protein that nucleates microtubule assembly, stabilizes the neuronal cytoskeleton, and supports axonal transport [1]. Alternative splicing of the single human *MAPT* gene generates six isoforms distinguished by zero to two N-terminal inserts (0N–2N) and three or four microtubule-binding repeats (3R–4R). Isoform expression is developmentally regulated, and individual isoforms localize to axonal, dendritic, nuclear, and synaptic compartments, indicating functions beyond microtubule stabilization, including RNA metabolism and genome maintenance [2]. Tau function is further modulated by extensive post-translational modifications (PTMs), such as phosphorylation, acetylation, ubiquitination, truncation, and others, that regulate its binding partners, subcellular localization, and turnover [3]. In Alzheimer’s disease (AD) and more than twenty related Tauopathies, aberrant hyperphosphorylation detaches Tau from microtubules, drives its somatodendritic and synaptic mis-localization, and promotes its assembly into paired helical filaments and neurofibrillary tangles, which closely correlate with synaptic loss and cognitive decline [4].

Mass-spectrometry–based proteomics has profoundly advanced our understanding of Tau’s interactome [5,6,7,8,9,10,11,12,13,14,15,16,17,18]. In post-mortem AD brains, laser-capture microdissection of neurofibrillary tangles followed by PHF1-directed co-immunoprecipitation identified high-confidence interactors that include vesicle-fusion factors (VAMP2, NSF), vacuolar ATPase subunits, and the kinases CAMK2A and CDK5 [6]. A complementary co-IP/MS comparison of total Tau from classical versus rapidly progressive AD cortices revealed additional interactors, with synaptic protein DPYSL4 and mitochondrial complex-III subunit UQCRC2 emerging as potential drivers of clinical variability [18]. In SH-SY5Y cells, GFP pull-down of full-length Tau showed enrichment for RNA-binding proteins, ribosomal subunits, chaperones, and proteasome components with the frontotemporal dementia (FTD) P301L mutation selectively perturbing C-terminal interactions [7]. More recently, APEX2-mediated proximity labeling in NGN2-induced human neurons mapped Tau associated proteins, spanning SNARE complex proteins, cytoskeletal regulators, and metabolic enzymes, and demonstrated that neuronal activity recruits Tau to presynaptic sites [14]. While these landmark studies define a comprehensive Tau interactome at discrete post-mortem or in vitro time points, they are not conducive to revealing the dynamic changes in the Tau interactome that are hypothesized to occur during the in vivo progression of Tauopathies in the CNS.

*Drosophila melanogaster* provides a uniquely tractable in vivo system for studying Tau toxicity and dysfunction with precise spatiotemporal control of transgenic human Tau expression [19,20,21,22,23,24,25]. In fact, panneuronal expression of human Tau via the UAS/Gal4 system [26] recapitulates key features of Tauopathies, including neurodegeneration, shortened lifespan, and associative learning deficits, in an isoform- and phosphorylation-dependent manner [19,20,21,23,24,25]. Using the TARGET (Temporal And Regional Gene Expression Targeting) modification of UAS/Gal4 [27,28] to restrict human Tau expression to the adult brain, we previously described the sequential appearance of disease-associated phospho-epitopes broadly predictive of phenotypic manifestations. In this setting, learning and memory impairments are apparent at 12 days of transgene expression, but not earlier and are followed by the onset of premature lethality a week or so later [19,20,21,22].

As the temporally specific appearance of pathological phenotypes in *Drosophila* broadly emulates Tauopathy progression in humans [19], we aimed to capture dynamic changes in the Tau interactome that may underlie neuronal dysfunction and deficits in learning and memory. Quantitative proteomics revealed that, during early transgene expression, Tau predominantly associates with cytosolic, cytoskeletal, and ribosomal proteins. By day 12, however, coincident with the onset of learning and memory impairments, Tau shows a preferential association with mitochondrial and synaptic proteins.

## 2. Results

### 2.1. Temporal Dynamics of Tau Interactome at 4, 8, and 12 Days Post-Induction

**Figure 1:**
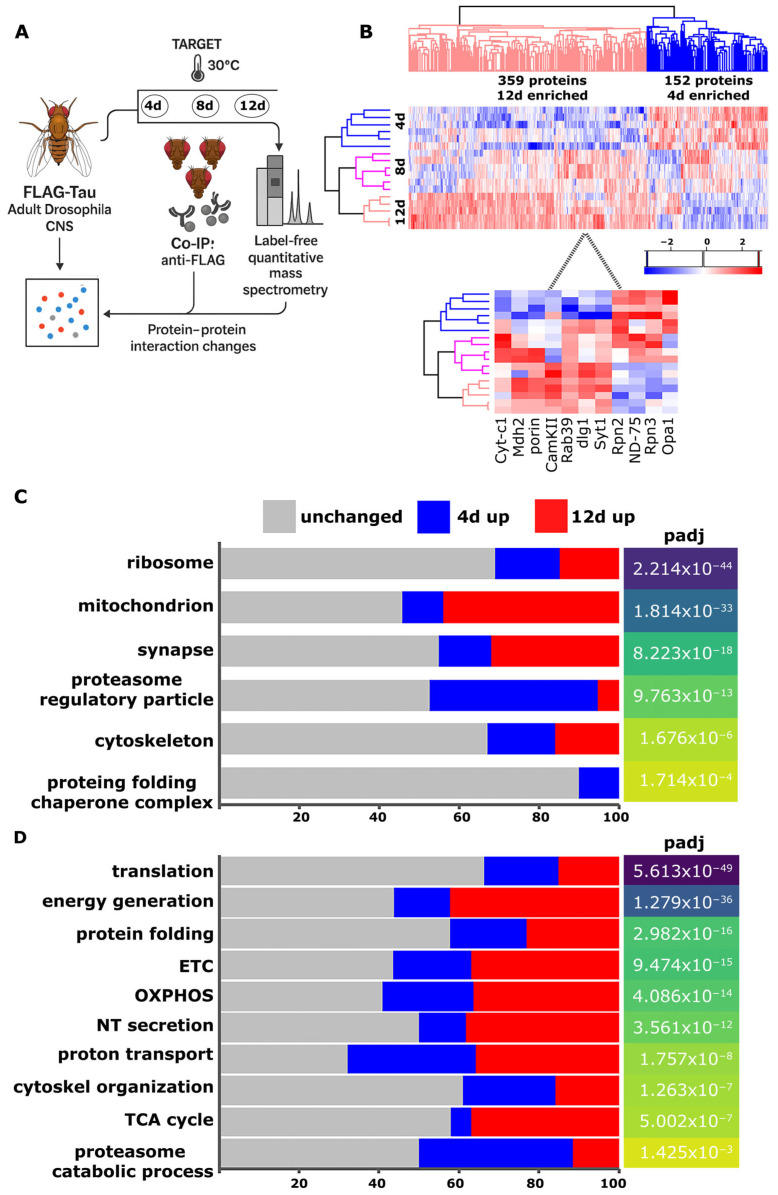
Temporal dynamics of the Tau interactome in adult Drosophila CNS. (**A**) Experimental workflow. FLAG-tagged 2N4R human Tau was expressed panneuronally in adult flies using the TARGET system (repressed at 18 °C, induced at 30 °C). Fly heads were harvested after 4, 8, or 12 days of transgene induction, Tau complexes isolated by anti-FLAG co-immunoprecipitation, and interacting proteins identified and quantified by label-free mass spectrometry. (**B**) Hierarchical clustering of temporally regulated Tau interactors. Of 1258 high-confidence clients, 541 showed significant time-dependent changes (FDR < 0.05). Early-enriched proteins (182; peak at day 4) are shown in blue, late-enriched proteins (359; peak at day 12) in red. Stably associated proteins are not shown but can be found in Table S1. The heat-map color scale bar indicates relative protein abundance: blue reflects decreased abundance and red denotes increased abundance over time. The inset displays representative line plots for key proteins illustrating their kinetics; see text for details. (**C**) GO Cellular Component enrichment. Major subcellular compartments associated with Tau (ribosome, mitochondrion, synapse, proteasome regulatory particle, cytoskeleton, and protein folding chaperone complex are plotted with the percentage of proteins unchanged (gray), enriched at day 4 (blue) or at day 12 (red), alongside adjusted *p*-values for each category. p_adj_ denotes the multiple-testing–corrected *p* value returned by g:Profiler for each enriched GO term. (**D**) GO Biological Process enrichment. Key processes: translation, generation of precursor metabolites and energy, protein folding, electron transport chain (ETC), oxidative phosphorylation (OXPHOS), neurotransmitter secretion, proton transmembrane transport, cytoskeletal organization, Tricarboxylic Acid (TCA) cycle, and proteasome-mediated ubiquitin dependent protein catabolic process, are similarly color-coded for temporal enrichment, with corresponding adjusted *p*-values (p_adj_).

To determine how Tau’s protein–protein interactions change over time, we induced expression of FLAG-tagged 2N4R Tau transgene in the adult Drosophila CNS and performed co-immunoprecipitation coupled with label-free quantitative mass spectrometry after 4, 8 and 12 days of transgene expression (Figure 1A). Across these three time points, we identified 1258 high-confidence Tau clients whose temporal abundance profiles fell into three categories: 717 (Table S1) proteins that remained stably associated throughout the duration of the experiment, 182 early enriched proteins that peaked significantly at day 4 (Table S1, early interactors), and 359 late-enriched proteins whose interaction with Tau increased by day 12 (Table S1, late interactors). The heat map in Figure 1B illustrates these 541 interactors that changed significantly over time (FDR < 0.05), with red indicating increased abundance and blue indicating decreased abundance. We included an intermediate 8-day time point to resolve the kinetics of these transitions. At this time, many interactors show intermediate abundances, occupying an inflection zone between ‘early’ and ‘late’ interaction patterns (Figure 1B). This mid-course sampling improves clustering fidelity in the heat map and better links proteome remodeling to downstream phenotypes.

To place these dynamic results in context, we performed Gene Ontology enrichment analysis for Cellular Component (Figure 1C) and Biological Process (Figure 1D) on the entire interactome. This analysis highlighted six major compartments: the ribosome, mitochondrion, synapse, cytoskeleton, proteasome complex and the chaperonin-containing protein-folding machinery (Figure 1C). Parallel enrichment of biological process terms associates each compartment with distinct functions such as translation, oxidative phosphorylation and metabolic energy production, neurotransmitter secretion, cytoskeleton organization, ubiquitin-dependent protein catabolic processes and protein folding (Figure 1D).

Although each compartment/process contains proteins from all three temporal clusters, their relative distributions vary, reflecting time-dependent associations with Tau (Figure 1C; gray, unchanged; blue, 4 d enriched; red, 12 d enriched). Ribosomal proteins are the most abundant (Figure 1C, p_adj_ reflects the significance of enrichment) and represent the largest class of stable Tau interactors, consistent with its persistent association with the translational machinery [29,30,31]. Within this compartment, many factors function in translation initiation (elF1, elF3a), elongation (eEF2, EEFγ), and co-translational quality control (Nacalpha, Rack1), reflecting active protein synthesis processes that may respond to Tau-induced proteostatic stress. A smaller subset of ribosomal partners peaked early at day 4 (small RpS and large RpL ribosomal subunit proteins), indicating an acute response to newly accumulated Tau (Table S2, Figure 1C,D).

Mitochondrial interactors were over-represented among the late-enriched proteins at day 12, exceeding their representation at day 4 and approaching enrichment seen in the stable cohort (Figure 1C, Table S2). The invariant mitochondrial cohort comprises core bioenergetic and quality-control components, including complex I subunits such as ND-19 and COX4, as well as metabolic enzymes Aldh and Ldh, indicating constitutive Tau engagement with basal mitochondrial machinery. Early responders at day 4 include ATP synthase subunits (ATPsynD, ATPsyndelta), complex I members ND-75 and ND-51, accessory factor ATPsynCF6, the peroxiredoxin Prx5, fusion regulator Opa1, Hsp70 paralog Hsc70-5, succinate dehydrogenase A (SdhA), mitoribosomal protein mRpL12 and the pyruvate dehydrogenase β-subunit Pdhb, suggesting acute perturbation of oxidative phosphorylation and mitochondrial dynamics immediately following human Tau expression. By day 12, Tau binds a broader array of tricarboxylic-acid cycle and electron transport chain factors such as malate dehydrogenase Mdh2, cytochrome c1 (Cyt-c1) and electron-transfer flavoprotein Etf-Qo consistent with mitochondrial dysfunction observed in Tauopathies (Figure 1D, Table S2). This progressive sequestration of mitochondrial components may underlie the bioenergetic deficits that exacerbate neuronal vulnerability as disease advances.

In the synaptic compartment, a core set of trafficking GTPases (Rab5, Rab7, Rab11), SNAREs (nSyb, Syx1A), adaptors (Dap160/Intersectin) and vesicle-coat proteins (Clc, Chc) remained bound at all three time points (Figure 1C, Table S2). These proteins drive vesicle trafficking, docking, and neurotransmitter release, underscoring the persistent association of Tau with synaptic transmission machinery. At day 4, Tau showed preferential association with active-zone assembly factors, notably the postsynaptic-density scaffold Prosap/Shank and the polarity kinase Par-1/MARK, which is known to phosphorylate Tau and enhance its toxicity in Drosophila neurons [32]. By day 12, the synaptic interactome became highly enriched and shifted to include regulators of vesicle fusion, Ca^2+^ homeostasis and plasticity, such as Rab39, Syngr, Atpα, Kcc, Snap25, Syt1, CaMKII, and the active-zone organizers CASK, Fife and Unc-13, implying progressive disruption of synaptic function (Figure 1C,D, Table S2) [33].

The cytoskeletal interactome was dominated by stable associations with structural and motor proteins (e.g., αTub85E, βTub97EF, Act5C, DAAM, DCTN2-p50, Khc-73, Unc-104 and Ank2) that maintain neuronal architecture and transport. These factors orchestrate cytoskeleton organization, filament assembly, and cargo movement (Figure 1C,D, Table S2). A subset of cytoskeletal modulators (Tsr, Zasp66, Eb1, Hts, Cher, Cam, Gel) peaked at day 4, indicating an acute cytoskeletal reorganization in response to early Tau accumulation. By day 12, Tau recruited additional cytoskeletal components including, motor proteins, filament regulators, scaffolds, and membrane-cytoskeleton linkers (Awd, Mhc, Dlg1, Chd64, Ran, Pnut, NinaC) consistent with late-stage cytoskeletal remodeling (Figure 1C,D and Table S2). Together, these sequential recruitment patterns delineate a dynamic cytoskeletal reconfiguration that correlates with the progressive Tau dependent toxicity [34,35].

Within the proteasome complex, Tau stably associated with core ATPase subunits (e.g., Rpt1, Rpt2, Rpt3, Rpt5, Rpt6) at all time points (Figure 1C,D, Table S2), whereas regulatory non-ATPase subunits (e.g., Rpn1, Rpn2, Rpn3) exhibited pronounced enrichment at day 4 that declined by day 12 (Figure 1C,D, Table S2). These subunits mediate ubiquitin-dependent protein degradation, highlighting the engagement of tau with proteostasis mechanisms. The transient early interaction with regulatory cap components may reflect attempts at Tau clearance, but progressive PTMs (e.g., phosphorylation) likely reduce its interaction with regulatory subunits while preserving affinity for the ATPase core. This shift may reduce the proteasomal processing of hyperphosphorylated Tau [36], contributing to its pathogenic accumulation.

Finally, within the chaperonin-containing TCP-1 complex (CCT/TRiC), Tau maintained stable association with all eight core subunits (CCT1–CCT8) and the CCT-interactor CG11999, indicating persistent engagement of the folding chamber (Figure 1C, Table S2). Conversely, interaction with Hsp83 (the Drosophila ortholog of Hsp90) [37] peaked at day 4 and declined by day 12 (Table S2). Hsp90 cooperates with co-chaperones to stabilize client proteins, drive conformational maturation, and facilitate release from upstream folding machinery, thereby maintaining proteome integrity and preventing aggregation [38,39]. Although our co-IP/MS experiment does not establish a mechanism, one working model is that Tau remains associated with CCT/TRiC while Hsp83 engagement diminishes, potentially reducing access to Hsp83-dependent folding cycles (for example as post-translational modifications accumulate) and thus promoting misfolding.

### 2.2. Tau Expression Impairs Mitochondrial Respiration and Alters Carbohydrate Homeostasis

**Figure 2:**
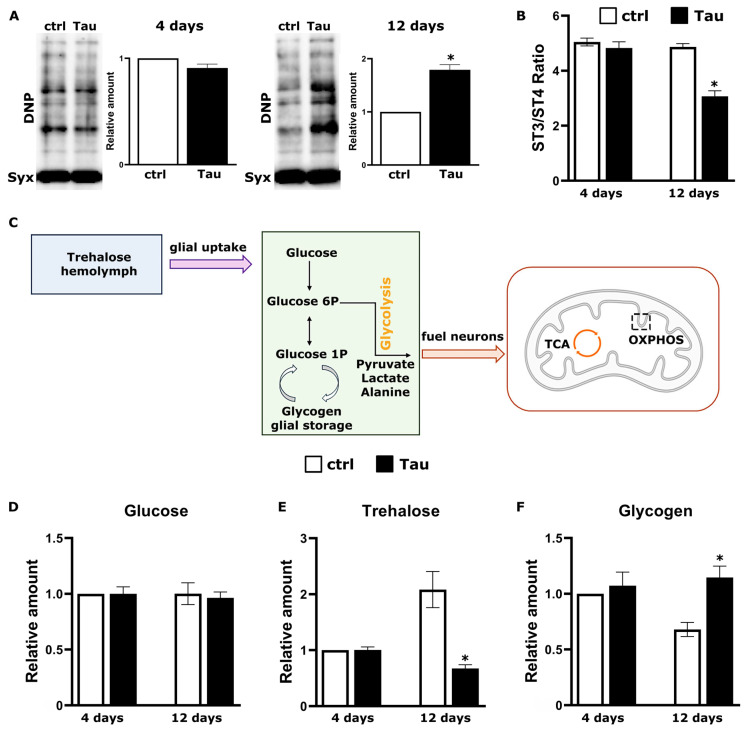
Tau overexpression impairs mitochondrial respiration and perturbs carbohydrate homeostasis in adult Drosophila CNS. (**A**) Protein carbonylation assessed by OxyBlot in head lysates from control (ctrl) and 2N4R Tau-expressing flies at 4 and 12 days post-induction, with Syntaxin (Syx) as a loading control. Controls are *elav*^C155^-GAL4; tub-Gal80^ts^ heterozygotes. Quantification shows relative carbonyl levels normalized to ctrl (mean ± SEM; *n* = 3, * *p* = 0.0002 versus ctrl). (**B**) Respiratory control ratio (State 3/State 4; RCR) measured in mitochondria isolated from heads at 4 and 12 days post-induction. White bars, ctrl; black bars, Tau (mean ± SEM; *n* = 4; * *p* = 0.0002 versus ctrl). (**C**) Cartoon of carbohydrate handling in the adult Drosophila brain. Trehalose from the hemolymph is taken up mainly by glia, hydrolyzed to glucose, and funneled through glycolysis to lactate/alanine that are shuttled to neurons to fuel mitochondrial oxidative phosphorylation (OXPHOS). Glia store glycogen and can mobilize it to buffer demand; under pathological stress, glycogen can also accumulate in neurons. (**D**) Glucose levels in head homogenates at 4 and 12 days post-induction (mean ± SEM; *n* = 6). No significant differences were observed between genotypes (*p* > 0.05). Levels are normalized to control 4 d. (**E**) Trehalose levels in head homogenates at 4 and 12 days post-induction, showing a failure of Tau-expressing flies to upregulate trehalose at day 12 (mean ± SEM; *n* = 5, * *p* = 0.00001 versus ctrl 12 d). Quantification shows relative levels normalized to ctrl 4 d. Control flies at 4 d of induction have lower trehalose levels than controls at 12 d (mean ± SEM; *n* = 5, *p* = 0.0003). (**F**) Glycogen levels in head homogenates at 4 and 12 days, demonstrating increased glycogen accumulation in Tau-expressing flies at day 12 (mean ± SEM; *n* = 6, * *p* = 0.0009 versus ctrl 12 d). Control flies at 12 d of induction have lower glycogen levels than those at 4 d (mean ± SEM; *n* = 6, *p* = 0.01). Data were analyzed using standard parametric statistics, with Student’s *t*-tests performed for each comparison.

Given the progressive enrichment of mitochondrial proteins in the Tau interactome by day 12 (Figure 1C,D), we hypothesized that Tau overexpression impairs mitochondrial bioenergetics in the adult Drosophila CNS. To assess oxidative damage, we performed OxyBlot analysis of carbonylated proteins in fly head lysates (Figure 2A). At 4 days, levels of protein carbonylation were similar between Tau-expressing and control flies, suggesting no detectable oxidative stress at this early time point. In contrast, by 12 days, Tau-expressing flies exhibited a marked increase in protein oxidation, consistent with impaired mitochondrial function and redox imbalance.

To evaluate mitochondrial function directly, we isolated mitochondria from adult fly heads at 4 and 12 days of Tau expression and measured oxygen consumption using a Clark-type electrode (Figure 2B). Respiration was recorded under basal (State 2), ADP-stimulated (State 3) and oligomycin-inhibited (State 4) conditions. The respiratory control ratio (RCR; State 3/State 4) served as a quantitative indicator of coupling efficiency and respiratory capacity [40]. At day 4, the RCR of mitochondria from 2N4R expressing flies was indistinguishable from that of *elav*^C155^-GAL4; tub-Gal80^ts^ heterozygote controls, indicating intact coupling (Figure 2B). By day 12, however, Tau-expressing mitochondria exhibited a significant reduction in RCR compared to controls. This impairment coincides with the proteomic enrichment of electron transport and tricarboxylic acid (TCA) cycle components among late-stage Tau interactors (Figure 1D, Table S2), implicating progressive disruption of mitochondrial bioenergetics by Tau overexpression.

To assess whether Tau-mediated mitochondrial dysfunction disrupts brain energy homeostasis, we next quantified key carbohydrates in fly heads at the same time points. In Drosophila, as in mammals, neuronal function depends heavily on glial uptake and metabolism of trehalose, the major sugar in the hemolymph, which is catabolized to lactate and alanine to fuel neurons via the glycolytic pathway (Figure 2C) [41]. In parallel, glycogen provides a longer-term glucose reserve, predominantly stored in glia but also accumulating in neurons under pathological conditions [42,43].

Glucose levels did not differ significantly between Tau-expressing and control flies at either time point (Figure 2D), indicating that glucose delivery to the CNS appears unaltered. In contrast, control flies exhibited age-associated increase in trehalose levels [44] at 12 days relative to that in 4 days, which is consistent with enhanced metabolic demand or storage. This increase was not observed in Tau-expressing flies (Figure 2E). Because whole-head trehalose reflects carbohydrate handling across glia and neurons, these patterns are consistent with altered glial carbohydrate utilization and glia–neuron metabolic coupling under Tau burden. Glycogen levels declined between days 4 and 12 in controls but were maintained in Tau-expressing flies, yielding significantly higher steady-state glycogen at day 12 (Figure 2F). This late-stage glycogen accumulation likely reflects a pathological shift in carbohydrate metabolism, in line with reports that aberrant neuronal glycogen deposition is linked to neurodegeneration and shortened lifespan in both Drosophila and mammalian models [45,46].

Collectively, these results demonstrate that human Tau expression progressively impairs mitochondrial respiration, increases oxidative damage and perturbs sugar metabolism, particularly trehalose utilization and glycogen storage, thereby exacerbating bioenergetic stress.

### 2.3. Porin Down-Regulation Disrupts Tau-Mitochondria Interaction and Alters Tau Biochemical Properties

Voltage-dependent anion channels (VDACs) are the most abundant proteins of the outer mitochondrial membrane. Their primary role is to mediate exchange of ions and metabolites between the cytosol and mitochondria [47]. In particular, VDAC1 helps regulate redox balance in both compartments [48]. Prior work in AD patient tissue and mouse models has shown that phosphorylated Tau can interact with VDAC1, potentially occluding the pore and impairing mitochondrial function [49,50,51]. Because our day-12 data showed disrupted redox homeostasis (Figure 2) and our proteomics indicated increased interaction between Tau and Porin (Figure 1B, Table S2), the Drosophila homolog of VDAC1, we next tested the physiological significance of this interaction. To do so, we used RNA interference (RNAi) to attenuate Porin expression in flies expressing human Tau (0N4R) panneuronally under *elav*^C155^-GAL4. For cross-isoform consistency, we also measured brain trehalose, glucose, and glycogen in the 0N4R background, which recapitulated the trends seen in Figure 2D–F and are provided in Supplementary Figure S1.

**Figure 3:**
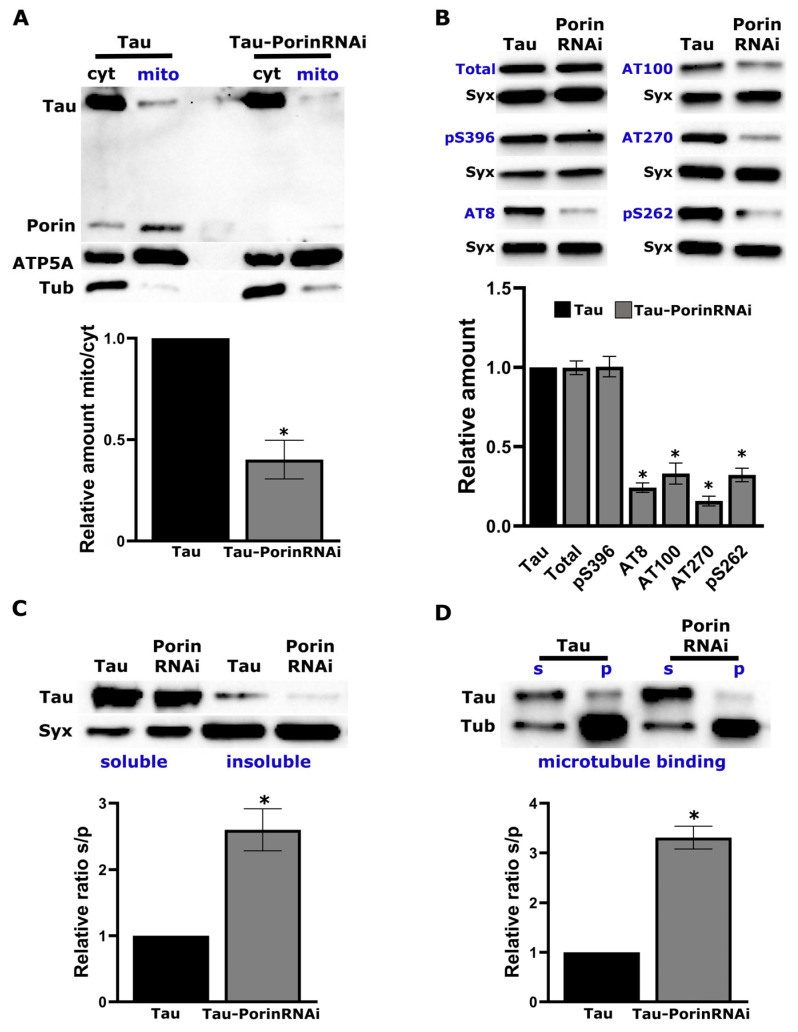
Porin downregulation reduces mitochondrial association of Tau and alters its phosphorylation, solubility, and microtubule binding. (**A**) Mitochondrial fractionation. Head lysates from adult flies expressing panneuronal human Tau alone (Tau) or together with Porin RNAi (Tau + Porin RNAi) were separated into cytosolic (Cyt) and mitochondrial (Mito) fractions. Western blots were probed for Tau, Porin, the mitochondrial marker ATP5A and the cytosolic marker Tubulin. Bar graph shows the ratio of mitochondrial to cytosolic Tau (mean ± SEM; *n* = 3; * *p* = 0.0033 versus Tau alone). (**B**) Phospho-Tau analysis. Whole-head lysates from Tau and Tau + Porin RNAi flies were immunoblotted with antibodies against total Tau and phospho-Tau epitopes: pS396, AT8 (pS202/pT205), AT100 (pT212/pS214), AT270 (pT181), and pS262. Syntaxin (Syx) served as loading control. Quantification shows phospho-Tau levels normalized to total Tau (mean ± SEM; *n* = 4; * *p* < 0.0001). (**C**) Solubility assay. Head lysates were fractionated into soluble (S) and insoluble (P) fractions and blotted for Tau and Syx. Bar graph depicts the ratio of soluble to insoluble Tau (mean ± SEM; *n* = 3; * *p* = 0.0073). (**D**) Microtubule-binding assay. Taxol-stabilized microtubules were incubated with head lysates, then pelleted (P) to isolate microtubule-bound proteins; the supernatant (S) contains unbound proteins. Blots were probed for Tau and Tubulin (Tub). Quantification shows the ratio of pelleted to supernatant Tau, reflecting microtubule association (mean ± SEM; *n* = 3; * *p* = 0.0005). The data were analyzed by standard parametric statistics using Dunnett’s tests relative to the designated control. Uncropped versions of the blots with molecular weight markers are provided in Figure S2.

In the RNAi condition, Porin was undetectable, confirming efficient knockdown, while ATP5A and Tubulin levels remained constant (Figure 3A). To determine whether Porin promotes Tau association with mitochondria, we isolated mitochondria from Tau-expressing adult fly heads, with and without Porin knockdown and quantified Tau levels in the mitochondrial fraction by Western blot. Although cytosolic Tau levels were similar across conditions, Tau co-precipitation with mitochondria was significantly reduced upon Porin depletion (relative amount mito/cyt, Figure 3A).

Given the established link between mitochondrial-localized Tau and its hyperphosphorylated state in disease [52,53,54], we next investigated whether Porin downregulation affects Tau phosphorylation (Figure 3B). Adult fly head lysates were analyzed by Western blot using a panel of phosphorylation-specific antibodies targeting epitopes commonly enriched in Alzheimer’s disease: AT270 (pT181), AT8 (pS202/pT205), AT100 (pT212/pS214), anti-pS262 and anti-pS396. Although total Tau levels remained unchanged, phosphorylation at all tested sites was markedly reduced following Porin knockdown, except for pS396, which paralleled total Tau levels (Figure 3B).

Because Tau aggregation into insoluble species is a key pathogenic event [4], we assessed whether Porin influences Tau solubility. Head lysates were fractionated into soluble and insoluble fractions and analyzed by immunoblotting for Tau. Porin downregulation resulted in a clear shift of Tau toward the soluble fraction, indicative of reduced aggregation propensity (Figure 3C).

Finally, we tested whether Porin downregulation alters the cytoskeletal properties of Tau. Given that Tau is a microtubule-associated protein, changes in its phosphorylation or solubility may affect its ability to bind microtubules [55]. We performed microtubule sedimentation assays on adult fly head lysates in the presence of Taxol to stabilize polymerized tubulin. Lysates were subjected to ultracentrifugation to separate microtubule-bound (pellet) from unbound (supernatant) proteins, and both fractions were analyzed by Western blot. As shown in Figure 3D, Porin knockdown led to a marked reduction in the amount of Tau recovered in the microtubule-bound fraction.

Collectively, these findings demonstrate that Porin modulates multiple biochemical and functional properties of Tau, including its association with mitochondria, phosphorylation at disease-relevant sites, aggregation state, and interaction with the microtubule cytoskeleton and underscore a physiologically significant interaction between Tau and Porin.

### 2.4. Porin Down-Regulation Potentiates Tau-Mediated Toxicity in Adult Neurons

Having established that Porin modulates multiple biochemical features of Tau, we next sought to determine whether Porin levels influence the in vivo toxicity of human Tau in adult neurons. All subsequent experiments were performed under the TARGET system to restrict expression to the adult nervous system. Flies were raised at 18 °C and shifted to 30 °C two days post-eclosion to induce Tau expression for 12 days.

**Figure 4:**
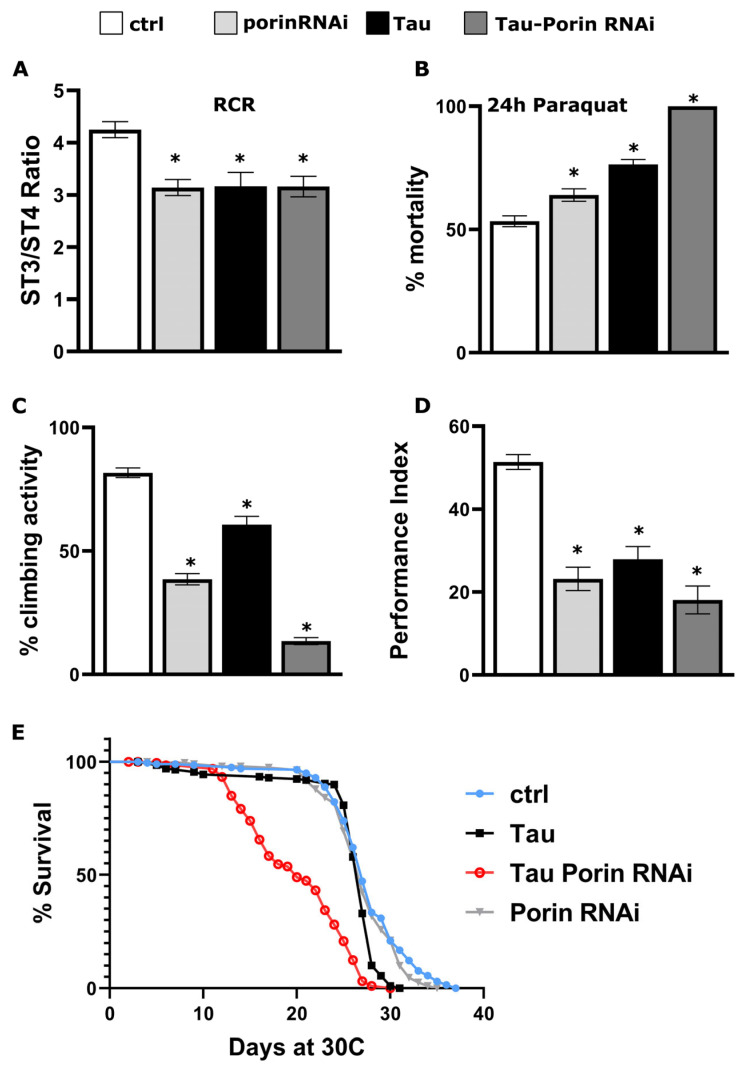
Porin downregulation exacerbates Tau-mediated neuronal dysfunction in adult Drosophila. (**A**) Respiratory control ratio (State 3/State 4) measured in mitochondria isolated from heads of adult flies after 12 days of induction. White bar, control (*elav*^C155^-GAL4; tub-Gal80^ts^ heterozygotes); light gray, flies expressing Porin RNAi alone; black, Tau expressing flies and dark gray, Tau and Porin RNAi co-expressing flies. All experimental genotypes show a significant reduction in coupling efficiency compared to control heterozygotes (mean ± SEM; *n* = 4; * *p* = 0.006 Dunnett’s versus ctrl). (**B**) Sensitivity to oxidative stress (*n* = 15): % mortality after 24 h exposure to 30 mM paraquat. Controls (white) exhibit ~50% mortality, which increases in Tau (black, * *p* < 0.0001 versus control), Porin RNAi (light gray, * *p* = 0.02 versus control), and reaches 100% in the double transgene (dark gray, * *p* < 0.0001 versus control). (**C**) Negative geotaxis (climbing) assay after 12 days of induction. Percentage of flies crossing the 66 mL mark of a 100 mL cylinder in 20 s (*n* = 10): control driver heterozygotes (white), Tau (black, * *p* < 0.0001 versus control), Porin RNAi (light gray, * *p* < 0.0001 versus control), and the double transgene (dark gray, * *p* < 0.0001 versus control) (**D**) Learning performance of animals accumulating panneuronally after 12 days of induction the indicated transgenes, compared with driver heterozygotes (white bar). Data are mean ± SEM; *n* > 8; * *p* < 0.05 significant differences from control. Detailed statistics can be found in Table S3. (**E**) Survival curves at 30 °C following adult-specific induction. Control (light blue) and Porin RNAi alone (gray) overlap (χ^2^ = 4.162, *p* = 0.0413). Tau-expressing flies (black) exhibit a shortened lifespan (χ^2^ = 26.4, *p* < 0.0001 compared to controls), and the Tau + Porin RNAi group (red) undergoes a sharp increase in mortality (χ^2^ = 147.8 and *p* < 0.0001 compared to Tau).

Given the previously demonstrated disruption of mitochondrial activity by Tau at 12 days of induction (Figure 2A), we first assessed mitochondrial respiration in isolated fly head mitochondria. Using a Clark-type oxygen electrode, we measured basal, ADP-stimulated, and oligomycin-inhibited respiration to calculate the respiratory control ratio (RCR). Compared to heterozygous *elav*^C155^-GAL4; tub-Gal80^ts^ controls, all three experimental genotypes, namely Tau alone, Porin RNAi alone, and Tau combined with Porin RNAi, exhibited significantly reduced RCRs at 12 days (Figure 4A). These results indicate that Porin downregulation, even in the absence of Tau, is sufficient to impair mitochondrial respiratory capacity.

Since oxidative stress is a hallmark of both mitochondrial dysfunction and Tau pathology, we next assessed sensitivity to exogenous reactive oxygen species by exposing flies to paraquat. In line with the respiration data (Figure 4A), all three experimental groups, Tau, Porin RNAi, and the double combination, displayed significantly increased paraquat sensitivity compared to controls (Figure 4B), further supporting a role for Porin in maintaining oxidative homeostasis under both physiological and pathological conditions. Notably, after 24 h of treatment all Tau and Porin-RNAi co-expressing flies had died (Figure 4B).

In parallel, we evaluated locomotor performance by measuring climbing ability after 12 days of induction. Tau expression alone resulted in a pronounced locomotor deficit (Figure 4C), consistent with previous reports of Tau-induced neuronal dysfunction [56]. Notably, this deficit was markedly exacerbated by Porin knockdown in Tau-expressing flies (Figure 4C). Moreover, Porin RNAi by itself also impaired climbing, indicating that Porin is required for adult neuronal function in this assay, even in the absence of Tau.

We next assessed short-term associative learning (3 min memory) using an olfactory classical conditioning paradigm. As previously reported, Tau-expressing flies trained 12 days post-induction performed significantly worse than controls in the 3 min memory test, indicating impaired learning (Figure 4D) [19,20]. Importantly, Porin downregulation did not ameliorate this defect. In fact, Porin RNAi alone also impaired learning, and Tau/Porin RNAi double transgene flies performed even worse than those expressing Tau alone (Figure 4D, detailed statistics in Table S3).

Because aversive olfactory conditioning requires arm traversal in a T-maze, we considered whether impaired performance might reflect motor limitations rather than learning deficit per se. To dissociate these, we quantified baseline locomotion under a lower-demand short-vial negative geotaxis assay. In this paradigm, single-transgenic cohorts (Tau alone or Porin RNAi alone) were indistinguishable from controls across all zones, whereas only the double-transgenic group showed a modest redistribution toward lower zones (Figure S3). These data indicate that baseline locomotion under a mild challenge is intact in Tau-only and Porin-only flies at the time learning was tested, arguing against a general motor confound for the learning impairments observed in each single-transgenic group (Figure 4D). Conversely, the high-demand climbing assay (negative geotaxis in a 100 mL cylinder, Figure 4C) is more sensitive to motor/energetic limitations and is impaired by Tau, by Porin RNAi, and most strongly by their combination, consistent with Porin’s role in mitochondrial capacity. Taken together, the pattern across assays suggests that the more-than-additive learning deficit in Tau-Porin RNAi is not explained solely by motility but is better aligned with bioenergetic stress impacting neuronal/synaptic function, in keeping with our respiration/oxidative-stress data and Porin’s contribution to mitochondrial homeostasis.

Finally, to determine whether Porin affects Tau-induced toxicity, we measured lifespan in flies expressing panneuronally Tau alone or in combination with Porin RNAi. As previously reported [20,57], panneuronal expression of Tau results in a progressive decline in survival (Figure 4E). However, co-expression of Tau and Porin RNAi markedly exacerbated mortality, with premature lethality evident before day 20 (Figure 4E). In contrast, the lifespan of Porin RNAi differed modestly from controls, indicating that the enhanced lethality requires the presence of Tau and is not a general consequence of Porin reduction.

Together, these findings indicate a dissociation between biochemical markers (reduced phosphorylation/aggregation) and functional outcomes (shorter lifespan, impaired mitochondrial respiration, increased oxidative stress, reduced locomotion, and learning deficits), suggesting that Porin supports neuronal resilience and mitochondrial homeostasis, even as it may facilitate specific Tau modifications. Consistent with this interpretation, Porin knockdown alone impaired mitochondrial and neuronal function, underscoring its role in regulating bioenergetic capacity and neurophysiological performance.

## 3. Discussion

Tauopathies are characterized by the progressive intracellular accumulation of aberrantly modified Tau protein, which drives synaptic dysfunction, neuronal loss and cognitive decline in AD and related disorders [55]. Although neurofibrillary tangles (NFTs) and synaptic degeneration characterize end-stage pathology, mounting evidence indicates that Tau-driven aberrations in molecular processes and pathways emerge long before clinical symptoms appear [58,59]. Over the past decade, proteomic approaches have extended our understanding of Tau beyond its canonical microtubule-binding function, revealing dynamic interactions with a diverse array of cellular machineries [12,60]. Affinity-purification mass spectrometry and in vivo crosslinking experiments have demonstrated that Tau engages classical heat-shock chaperones (Hsp70/Hsp90) and the 20S proteasome, underscoring its regulation by proteostasis networks, as well as ribonucleoprotein complexes involved in RNA splicing and translation [9,15,18]. Complementary phospho-specific interactome mapping has revealed that the phosphorylation status of Tau reshapes profoundly its binding landscape. Early appearing phospho-epitopes such as pT217 recruit E3 ubiquitin ligase adaptors and the autophagy receptor SQSTM1, indicating finely tuned phosphorylation-dependent degradation pathways [10]. At advanced disease stages, hyperphosphorylated Tau sequesters synaptic vesicle-associated proteins and endolysosomal regulators, offering a molecular explanation for synaptic dysfunction and impaired vesicle trafficking in AD [6,14]. However, these studies have not yet addressed the temporal sequence by which Tau engages distinct cellular components and molecular mechanisms during disease progression.

By applying quantitative label-free proteomics to adult *Drosophila* brains at 4, 8, and 12 days following Tau induction, we resolved a coherent temporal cascade of Tau cellular interactions. We selected 4, 8, and 12 days post-induction to align mechanistically with established phenotypic milestones in this adult-onset model. Prior work shows that learning and memory impairments first emerge at 12 d, followed by premature lethality, whereas earlier days capture pre-symptomatic molecular remodeling [20,21,22]. Accordingly, 12 d anchors the functional onset, 4 d samples the early proteostatic/translational response, and the 8 d midpoint improves clustering fidelity and resolves transition kinetics between “early” and “late” interactors in our co-IP/MS heatmap (Figure 1B), allowing us to link interactome shifts to downstream phenotypes. Because co-IP/MS captures both direct and indirect interactors and reports relative abundance, our temporal associations should be interpreted as changes in complex composition rather than proven direct binding. At day 4, Tau associated predominantly with ribosomal proteins, RNA-binding factors, and chaperones, consistent with an early proteostatic/translational response. By day 8, many interactors exhibited intermediate abundances, marking a transition between early and late clusters (Figure 1B). By day 12, coincident with the onset of learning and memory impairments [19,20], Tau showed a preferential association with mitochondrial and synaptic proteins, in line with GO enrichment for bioenergetics and synaptic processes (Figure 1C,D; Table S2).

Mitochondrial dysfunction is now recognized as a pivotal driver of Tauopathy progression. As the organelles responsible for ATP synthesis, Ca^2+^ buffering, and reactive-oxygen–species homeostasis, mitochondria in Alzheimer’s disease consistently exhibit altered morphology (including abnormal size and cristae disruption), defective oxidative phosphorylation, increased ROS production, impaired biogenesis, and changes in mitochondrial mass, enzyme activities, and mtDNA integrity. These deficits arise in both familial and sporadic AD models and patient samples, underscoring their fundamental role in pathogenesis [61,62,63]. Hyperphosphorylated Tau has been shown to cause mitochondrial damage by disturbing mitochondrial dynamics [64] and reducing the expression of components of the mitochondrial respiratory chain complex and antioxidant enzymes [65]. Several cellular models and animal models carrying mostly FTDP-17 associated mutations (i.e., P301L, P301S, R406W) have revealed aberrations in mitochondrial function caused by the pathological accumulation of Tau. Disrupted activity and altered composition of mitochondrial enzymes were detected in the P301S mouse model of Tauopathy [66]. Overexpression of the P301L mutant human Tau protein was reported to decrease ATP levels, increase susceptibility to oxidative stress and impair mitophagy in cultured neuroblastoma cell [67,68]. Finally, mice expressing P301L mutant exhibit reduced levels of mitochondrial complex V in affected brain regions, and a similar mitochondrial deficit was evident in brain tissue from patients harboring the same mutation [65].

In our *Drosophila* model, Tau accumulation triggers a sequential impairment of bioenergetic homeostasis that parallels cognitive decline and the ordered appearance of disease-associated phospho-epitopes [19,20]. At 4 days post-induction, the mitochondrial respiratory control ratio (State 3/State 4) remained intact but declined significantly by day 12, accompanied by increased protein carbonylation, indicating the onset of oxidative stress and impaired bioenergetics. Parallel CNS metabolite analyses showed that, unlike controls, Tau-expressing flies failed to upregulate trehalose at day 12 and instead accumulated glycogen, a phenomenon linked to pathological neuronal glycogen overload in both insect and mammalian systems [42,45,46]. These findings are consistent with reports of reduced glucose uptake, attenuated glycolytic flux, and disrupted glia–neuron metabolic coupling in Tau transgenic mice and in tissues from patients with AD [63,69,70], suggesting that disturbances in carbohydrate homeostasis may exacerbate the energy deficits imposed by mitochondrial dysfunction.

Porin/VDAC1, the voltage-dependent anion channel of the outer mitochondrial membrane, emerged as a late-stage Tau interactor. This finding paralleled observations in human AD brains and transgenic mouse models, in which VDAC1 levels increased and phosphorylated Tau bound directly to VDAC1, leading to elevated mitochondrial membrane permeability, impaired ATP/ADP exchange, and disrupted Ca^2+^ homeostasis [49,50]. Recent work with isolated brain mitochondria shows that phosphorylated Tau oligomers have no significant direct effect on electron-transport chain efficiency, pointing instead to indirect mechanisms, such as Tau engagement of VDAC1 at the outer membrane, to explain Tau-linked bioenergetic failure [71]. Consistent with a dose-dependent role, heterozygous VDAC1 reduction (VDAC1^+^/^−^/Tau mice) restores mitophagy, reduces oxidative damage, and preserves synaptic proteins (PSD95, synaptophysin, SNAP25) despite Tau overexpression [51].

Our data reveal a dissociation between Tau biochemistry and organismal outcomes when Porin/VDAC1 is depleted. Porin knockdown lowers Tau association with mitochondria and reduces disease-relevant phospho-epitopes and insolubility (Figure 3), yet respiration, oxidative-stress tolerance, locomotion, and learning all worsen (Figure 4). Notably, Porin RNAi alone impairs performance, indicating that Porin is required for baseline neuronal bioenergetics. We propose a simple model with two separable effects: (i) Porin loss reduces Tau–mitochondria association and lowers phospho-Tau and insolubility; (ii) because Porin is the principal channel for small metabolites, its depletion restricts energy and redox exchange at mitochondria. The energetic cost of the second effect outweighs the biochemical gains of the first, leading to worse functional phenotypes under Tau burden.

In summary, our temporal interactome analysis reveals a sequential vulnerability cascade, raising the possibility that early enhancement of proteostasis and mitochondrial function could delay synaptic degeneration and cognitive decline in Tauopathies.

## 4. Materials and Methods

### 4.1. Drosophila Culture and Strains

*Drosophila melanogaster* stocks and crosses were maintained on standard sugar-wheat-flour medium supplemented with soy flour and CaCl_2_ [23]. Panneuronal expression was driven by *elav*^C155^-GAL4 [72]. Unless stated otherwise, all crosses and experiments were performed at 25 °C. The *elav*^C155^-GAL4; tub-Gal80^ts^ line was generated using standard genetic techniques [73].

All experiments were performed with 4R Tau isoforms. The IP–MS pulldown used FLAG-tagged 2N4R, whereas the genetic interaction tests with Porin used 0N4R, our standard fly line. The UAS-htau0N4R was a gift from Dr. S. Thor (Linköping University) [25] and the Porin RNAi line was obtained from the Bloomington Stock Center (#BL29572). The construction of UAS-htauFLAG-2N4R has been described previously [24,74]. Double-transgenic combinations of hTau0N4R and Porin RNAi constructs were generated by standard genetic crosses.

### 4.2. LC/MS Analysis

The method is described in detail in [74]. Three biological replicates were collected for each genotype, each analyzed with two technical replicates. Each biological replicate was prepared from 15 mL of adult flies, yielding ~1.5 mL packed fly heads. Flies carrying the UAS-htauFLAG-2N4R transgene under panneuronal control of *elav*^C155^-GAL4; tub-Gal80^ts^ were maintained at 18 °C. Adult-specific expression was induced by transferring newly eclosed adults to 30 °C for 4, 8, or 12 days. Flies were then snap-frozen and decapitated by sieving in liquid N_2_. Heads were homogenized in lysis buffer (50 mM Tris-HCl, pH 7.4; 150 mM NaCl; 1 mM EDTA; 1% Triton X-100; protease and phosphatase inhibitors). Clarified lysates were incubated overnight at 4 °C with anti-FLAG agarose (Sigma-Aldrich, St. Louis, MO, USA). Lysates were processed by filter-aided sample preparation (FASP) with 10 kDa molecular-weight cut-off spin filters (VN01H02, Sartorius, Göttingen, Germany) [75]. Proteins were then alkylated and digested with 1 µg of mass-spectrometry-grade trypsin/Lys-C mix (Promega, Madison, WI, USA). Peptides were separated by nano-LC-MS/MS (liquid chromatography with tandem mass spectrometry) using an LTQ Orbitrap XL mass spectrometer (Thermo Fisher Scientific, Waltham, MA, USA) coupled to a nano-LC high performance liquid chromatography (RSLCnano, Thermo Fisher Scientific). Raw files were processed in MaxQuant v1.6.17.0 [76] with the Andromeda search engine against the *D. melanogaster* UniProt proteome (downloaded 17 February 2020; 22,045 entries) and the MaxQuant contaminants database. Protein abundance was reported as LFQ intensity. The log2-transformed LFQ intensities for all quantified protein groups, exported directly from MaxQuant are provided as an Excel file Supplementary Table S4. Differential abundance was assessed in Perseus v1.6.15.0 [77] using multifactor ANOVA with an FDR of 0.05. Statistically significant proteins were z-score–normalized and hierarchically clustered (average linkage; Euclidean distance), then visualized as heatmaps. Gene-Ontology analysis was performed with g:Profiler [78], with default multiple-testing correction, reporting adjusted *p*-values (p_adj_). Because many GO categories are tested simultaneously, g:Profiler adjusts raw *p*-values to control false positives. Thus, p_adj_ reflects the significance of enrichment after accounting for all terms tested; we consider terms with p_adj_ < 0.05 significant.

### 4.3. Western Blotting and Antibodies

For total-protein extraction, heads from adult flies (1–3 days post-eclosion) were homogenized in 1× Laemmli buffer (50 mM Tris-HCl, pH 6.8, 5% β-mercaptoethanol, 2% SDS, 10% glycerol, 0.01% bromophenol blue).

For mitochondrial isolation, 30 heads were homogenized on ice in isolation buffer (0.32 M sucrose, 10 mM EDTA, 10 mM Tris-HCl, pH 7.3) containing 2% (*w*/*v*) BSA. Homogenates were passed through gauze, which was then rinsed with additional isolation buffer to a final volume of 1.5 mL. After centrifugation for 10 min at 2200× *g*, the pellet (mitochondria) was resuspended in 1× Laemmli buffer. The supernatant, representing the mitochondria-free cytosol, was retained and supplemented with 5× Laemmli buffer.

All lysates were heated for 3 min at 95 °C, clarified at 11,000× *g* for 5 min, and the proteins resolved on 10% SDS–PAGE. PVDF membranes were probed with the following primary antibodies (dilutions in parentheses): Tau 5A6 (1:1000; Developmental Studies Hybridoma Bank, Iowa City, IA, USA), Tubulin E7 (1:1000; Developmental Studies Hybridoma Bank), AT100, AT270, and AT8 (each 1:1000; Thermo Fisher Scientific), anti-pS262 Tau (1:1000; Pro-Sci), anti-pS396 Tau (1:1000; Cell Signaling Technology, Danvers, MA, USA), anti-Porin/VDAC1 [20B12AF2] (1:1000; Abcam, Cambridge, UK), and anti-ATP5A [15H4C4] (1:2000; Abcam).

HRP-conjugated goat anti-mouse IgG and goat anti-rabbit IgG secondary antibodies (each 1:5000; Jackson ImmunoResearch, West Grove, PA, USA) were used. Membranes were co-probed with anti-Syntaxin 8C3 (1:3000; Developmental Studies Hybridoma Bank) as a loading control.

### 4.4. Oxyblot

For protein carbonylation analysis, tissue samples comprising ten heads were homogenized on ice in NP-40 lysis buffer (0.1% Nonidet P-40, 150 mM NaCl, 50 mM Tris-HCl, pH 8.0) supplemented with a protease-inhibitor cocktail (Sigma-Aldrich). Homogenates were centrifuged at 19,000× *g* for 10 min at 4 °C to obtain clear lysates. Protein concentrations were determined with the Bradford assay (Bio-Rad, Hercules, CA, USA).

Protein carbonyls were detected with the OxyBlot™ Protein Oxidation Detection Kit (Millipore, Burlington, MA, USA) following the manufacturer’s instructions with minor modifications. Briefly, 10 µg of protein was transferred to 0.5–1.5 mL microcentrifuge tubes and denatured by adding an equal volume of 12% SDS, giving a final SDS concentration of 6%. Derivatization was initiated by adding two volumes of 1× DNPH solution and incubating at room temperature for exactly 15 min, which maximizes derivatization while minimizing non-specific side reactions that arise with longer incubations (>30 min). The reaction was stopped by adding 1.5 volumes of neutralization solution; when reduction was required, 2-mercaptoethanol was included to a final concentration of 0.74 M.

Derivatized samples were resolved on 10% SDS–polyacrylamide gels and transferred to PVDF membranes. Membranes were probed with an anti-DNP antibody (1:150; supplied with the kit) to detect carbonyl groups and with the 8C3 antibody (1:3000) as a loading control.

### 4.5. Microtubule Binding Assay

Tau association with exogenous bovine microtubules was determined as described in [74]. Fifty heads were homogenized in 175 µL lysis buffer (80 mM PIPES pH 7.0, 2 mM MgCl_2_, 0.5 mM EGTA) supplemented with protease and phosphatase inhibitors. The homogenate was cleared by centrifugation at 4000× *g* for 5 min, followed by 7500× *g* for 20 min. Taxol (20 µM) was added to the supernatant, which was then incubated for 30 min at room temperature with 10 µL of paclitaxel-stabilized, in vitro-polymerized microtubules (1 mg/mL, Cytoskeleton, Denver, CO, USA). The mixture was layered onto 300 µL of 50% glycerol in binding buffer (80 mM PIPES pH 7.0, 2 mM MgCl_2_, 0.5 mM EGTA, 20 µM Taxol) and centrifuged at 110,000× *g* for 45 min. Supernatant and pellet fractions were collected, and Tau bound to microtubules was quantified by immunoblotting with Tau 5A6 and Tubulin E7 antibodies.

### 4.6. Tau Solubility Assay

Tau solubility was assessed following [21]. Ten fly heads were homogenized in 100 µL buffer (50 mM Tris–HCl pH 7.4, 175 mM NaCl, 1 M sucrose, 5 mM EDTA) containing protease and phosphatase inhibitors. The homogenate was cleared at 1000× *g* for 2 min, and the supernatant was centrifuged at 186,000× *g* for 2 h at 4 °C to yield the soluble S1 fraction. The pellet was resuspended in SDS buffer (50 mM Tris–HCl, pH 7.4; 175 mM NaCl; 5% SDS) and spun at 200,000× *g* for 2 h at 25 °C; the supernatant constituted the SDS-soluble S2 fraction. S1 and S2 were mixed with 2× Laemmli buffer, boiled for 5 min, resolved by SDS-PAGE, and immunoblotted with 5A6 and 8C3 antibodies.

### 4.7. Mitochondrial Respiration

Mitochondrial respiration was assessed as described in [40]. Mitochondria were isolated from 100 fly heads by mechanical homogenization on ice in mitochondrial isolation buffer (0.32 M sucrose, 10 mM EDTA, 10 mM Tris-HCl, pH 7.3) supplemented with 2% (*w*/*v*) bovine serum albumin (BSA). The homogenate was passed through sterile gauze to remove debris and centrifuged at 2200× *g* for 10 min at 4 °C. The resulting pellet was washed twice with BSA-free isolation buffer and resuspended in 200 µL of the same buffer. Protein concentration was determined with the Bradford assay.

Respiration was measured at 25 °C using a Clark-type oxygen electrode (Hansatech Instruments, King’s Lynn, UK). For each run, 150 µg of mitochondrial protein was added to 300 µL of respiration buffer (120 mM KCl, 5 mM KH_2_PO_4_, 3 mM HEPES, 1 mM EGTA, 1 mM MgCl_2_, 0.2% BSA, pH 7.2) containing 5 mM glutamate and 2.5 mM malate as substrates. Oxygen-consumption states were recorded sequentially as follows: State 2, representing basal oxygen consumption; State 3, ADP-stimulated respiration after the addition of 500 µM ADP; State 4, oligomycin-inhibited respiration after 6 µM oligomycin; and finally, the maximal uncoupled rate induced by 100 nM FCCP. The respiratory-control ratio (RCR) was calculated as the rate in State 3 divided by the rate in State 4 (ST3/ST4). All measurements were performed in triplicate with independent mitochondrial preparations.

### 4.8. Carbohydrate Quantification

Carbohydrate content in fly heads was measured as described previously [40]. Briefly, heads from ten adult flies (five males and five females) were homogenized on ice in phosphate-buffered saline (PBS) for glucose (GLU) and glycogen (GLY) assays, or in trehalase buffer (5 mM Tris-HCl, pH 6.6, 137 mM NaCl, 2.7 mM KCl) for trehalose (TREH) determination. Homogenates were centrifuged at 1200× *g* to remove debris, and the supernatants were heated at 70 °C for 10 min to inactivate endogenous enzymes. After a second centrifugation at maximum speed for 3 min, 30 µL of clarified supernatant (diluted 1:4 for GLU and GLY, undiluted for TREH) was dispensed into a 96-well plate.

Glucose was assayed by adding 100 µL of glucose reagent (Sigma-Aldrich) and incubating at 37 °C for 30 min. Glycogen was quantified in parallel reactions containing 1 U of amyloglucosidase (Sigma-Aldrich) to hydrolyze glycogen to glucose. Trehalose was measured by incubating samples with 100 µL of the glucose reagent in the presence or absence of 0.05 U mL^−1^ trehalase (Sigma-Aldrich) for 18 h at 37 °C. Absorbance was read at 540 nm. Trehalose and glycogen concentrations were calculated by subtracting baseline glucose values from the total glucose released after enzymatic digestion. Each experiment included at least three biological replicates per genotype or condition.

### 4.9. Paraquat Sensitivity

To assess paraquat sensitivity, flies carrying UAS-htau0N4R and/or UAS-Porin RNAi under *elav*^C155^-GAL4; tub-Gal80^ts^ were raised at 18 °C, then shifted to 30 °C for 12 days after eclosion to induce adult-specific expression. Paraquat feeding was carried out as previously described at 30 °C [57,74], using groups of 20 flies (10 males and 10 females) provided with standard food supplemented with 30 mM methyl viologen (Acros Organics, Geel, Belgium). At least 300 flies per genotype were scored.

### 4.10. Climbing Assay

Flies carrying UAS-htau0N4R and/or UAS-Porin RNAi under *elav*^C155^-GAL4; tub-Gal80^ts^ were raised at 18 °C, then shifted to 30 °C for 12 days after eclosion to induce adult-specific expression. Locomotor performance was assayed two ways as described previously [25,79]. For the primary, higher-demand test, 30 adult flies (15 males and 15 females) were placed in a 100 mL graduated cylinder marked at 66 mL. After gently tapping the flies to the bottom, the number that climbed past the 66 mL mark within 20 s was recorded. At least 300 flies per genotype were scored.

Alternatively, flies were gently transferred into an empty glass vial (2 cm diameter) held at 25 °C and 40% relative humidity under dim red illumination, then allowed to acclimate for 1 min. The vial was visually divided into three 2 cm zones (lower, middle, upper). Following a gentle tap to the vial’s base, negative geotaxis was recorded with a Basler AG acA1920-155 um camera at 1 frame/s. Nine seconds after tapping, the number of flies in each zone was counted, and zone frequency was calculated as the number of flies in that zone divided by the total in the group. Climbing performance was analyzed by Dunnett’s test relative to control driver heterozygotes.

### 4.11. Lifespan Determination

For longevity assessment [57], transgenic Drosophila co-expressing Tau and Porin RNAi under the control of *elav*^C155^-GAL4; tub-Gal80^ts^, along with single-copy driver controls, were maintained at 18 °C during development. Newly eclosed adults (1–3 days post-eclosion) were collected in cohorts of 20 flies (10 males and 10 females) and transferred to 30 °C to induce transgene expression. Mortality was recorded daily, with flies transferred to fresh food medium every 72 h to maintain optimal nutritional conditions. Each experimental group consisted of ≥200 individuals to ensure statistical power.

### 4.12. Behavioral Analysis

Animals expressing UAS-htau0N4R and UAS-Porin RNAi single and double transgenes under the control of the panneuronal *elav*^C155^-GAL4; tub-Gal80^ts^ driver were raised at 18 °C together with control single copy driver flies. Conditional transgene expression under this driver was induced specifically in adult flies by incubation at 30 °C for 12 days post-emergence. Progeny were collected in cohorts of 50–70 mixed-sex adults and subjected to classical olfactory-aversive conditioning [74,80]. Benzaldehyde (6% *v*/*v*) and 3-octanol (50% *v*/*v*) in isopropyl myristate (Fluka, Buchs, Switzerland) served as conditioned odors. Training and testing were conducted at 25 °C, 75% relative humidity, under dim red illumination. All genotypes were evaluated in parallel each day to ensure balanced experimental conditions.

### 4.13. Statistical Analysis

All quantifications of Western blots were carried out by densitometry, with each band’s intensity normalized to its corresponding protein reference control. The mean ratio for the control genotype was defined as 1.0, and all other values are expressed relative to that baseline. Data from at least three independent experiments are presented as mean ± SEM. Statistical comparisons against the control were made using Dunnett’s test. Similarly, measures of survival following oxidative-stress exposure were evaluated against control using Dunnett’s. Longevity data were analyzed using Kaplan–Meier method in GraphPad Prism 8.0.1; group differences were tested with the log-rank (Mantel–Cox) test. Finally, genotype-specific learning performance indices were tested by one-way ANOVA, followed by planned multiple comparisons using the least-squares means (LSM) approach. All parametric analyses were performed in JMP 7.0.1 (SAS Institute Inc., Cary, NC, USA) as previously described [74,81].

## Data Availability

Proteomics datasets used in this study are available from the corresponding author upon request.

## Supplementary Materials

The following supporting information can be downloaded at: https://www.mdpi.com/article/10.3390/ijms26199741/s1.
