# Supplementary material for: Temporal Interactome Mapping of Human Tau in Drosophila Reveals Progressive Mitochondrial Engagement and Porin/VDAC1-Dependent Modulation of Toxicity
Source: Int J Mol Sci. 2025 Oct 7;26(19):9741. doi: 10.3390/ijms26199741 (PMC12524354; doi:10.3390/ijms26199741)
Supplement: Supplementary file 1 [file ijms-26-09741-s001.zip › Supplemental Information.pdf]

Table S1. Tau interactome proteins (alphabetical order) and temporal class

	Gene	p-value	Gene	p-value	Gene	p-value
1	128up	0.803	14-3-3zeta	0.03245	Actn	0.02737
2	14-3-3epsilon	0.136	152078_at	0.00225	Adi1	0.00018
3	Acadvl	0.527	ABCB7	0.010315	Agxt	0.00013
4	Acbp2	0.18	Acat1	0.0088	Ak1	0.0118
5	Acer	0.9	ACC	0.000016	Alg-2	0.0379
6	Ack	0.761	AcCoAS	0.001879	alphaSnap	0.03574
7	Acp65Aa	0.127	Ace	0.002264	alpha-Spec	0.00264
8	ACSL1	0.379	Acsf2	0.004756	ALT	0.01027
9	Act57B	0.346	Acsl	0.006051	Amph	0.00274
10	Act5C	0.123	Act87E	0.039713	Argl	0.00088
11	Act79B	0.268	Adh	0.00135	Arr1	0.00319
12	Adk2	0.405	Adk2	0.023083	Arr2	0.03281
13	Adss	0.727	Agl	0.000455	ATPsynbeta	4.98e-06
14	Afg3l2	0.572	AGO1	0.018537	ATPsynCF6	0.00002
15	AGBE	0.325	AhcyL1	0.043855	ATPsynD	0.00374
16	Agpat3	0.408	Ald1	0.014861	ATPsyndelta	0.00772
17	Ahcy	0.115	alphaCOP	0.007418	ATPsynO	0.00015
18	Akap200	0.63	amon	0.001082	B52	0.04317
19	Akr1B	0.073	AnxB10	0.007704	blw	0.0019
20	AlaRS	0.674	AOX3	0.002324	Caf1-55	0.01993
21	Aldh	0.333	apolpp	0.000014	Cam	0.00023
22	Aldh7A1	0.166	Apt1	0.017778	cathD	0.04711
23	Aldh-III	0.383	Ar2	0.015442	Cbp53E	0.01649
24	ALiX	0.091	aralar1	0.00132	CG10031	0.00121
25	alpha-Cat	0.532	Arf4	0.04458	CG10799	0.00044
26	alpha-Est7	0.62	Argk1	0.001487	CG10984	0.03723
27	alpha-KGDHC	0.92	asrij	0.052768	CG11334	0.01029
28	alphaTub84B	0.09	Atpalpha	0.00275	CG11899	0.01286
29	alphaTub85E	0.172	ATPCL	0.025879	CG13367	0.00002
30	AMPdeam	0.703	ATPsynB	0.016873	CG1440	0.00622
31	Amy-p	0.547	awd	3.66e-06	CG15717	0.01912
32	Ank	0.677	BaraA2	0.001371	CG1737	7.28e-06
33	Ank2	0.497	Bbd	0.030034	CG2145	0.00759
34	AnxB9	0.098	BckdhB	0.052058	CG30022	0.00385
35	Aos1	0.205	bdl	0.042798	CG31997	0.02532
36	AP-1-2beta	0.188	Bet5	0.007179	CG44195	0.00041
37	AP-2alpha	0.724	bic	0.042196	CG5174	0.02306
38	AP-2mu	0.065	bor	0.001342	CG5793	0.00007
39	AP-2sigma	0.638	cact	0.005891	CG5973	0.00811
40	apt	0.103	CAH1	0.014229	CG6028	0.00594
41	Arf1	0.246	Calr	0.010381	CG6163	0.00186
42	Arl8	0.057	CaMKII	0.000204	CG7135	0.00804
43	Arp1	0.087	CanB	0.025174	CG7239	0.00024
44	AsnRS	0.237	CASK	0.020679	CG9391	0.00012
45	aspr	0.572	Cat	0.000547	CG9498	0.00259
46	Atat	0.337	CatB	0.043704	cher	3.67e-06

47	atl	0.516	cer	0.000447	Chro	5.97e-07
48	ATPsynE	0.403	CG10237	0.003469	CkIIalpha	0.00999
49	ATPsynF	0.289	CG10513	1.55e-06	COX5B	0.00612
50	ATPsynG	0.205	CG10527	0.02816	Cph	0.0018
51	ATPsyngamma	0.605	CG12163	0.000231	Cpr66D	2.06e-06
52	Bacc	0.329	CG12170	0.005139	Cpr72Ec	0.00427
53	Bap111	0.408	CG13151	0.021325	Crys	0.016
54	Bap55	0.342	CG13369	0.020492	CT7856	0.01434
55	BckdhA	0.462	CG14091	0.004205	Dip-B	0.01013
56	BEAF-32	0.398	CG14762-RA	0.00095	Dip-C	0.01456
57	ben	0.817	CG15093	0.000468	Dis3	0.00942
58	bero	0.1	CG15118	0.005476	dre4	7.64e-08
59	betaCOP	0.089	CG1545	0.015571	Droj2	0.00011
60	beta'COP	0.346	CG15894	0.001624	ebi	0.03129
61	beta-Spec	0.08	CG16760	0.000045	eEF1alpha1	0.00045
62	betaTub56D	0.166	CG16985	0.026181	eEF1alpha2	0.00044
63	betaTub97EF	0.172	CG17065	0.013777	eEF1beta	0.00508
64	Blos2	0.207	CG17162	0.009878	eIF2alpha	0.01993
65	bnb	0.685	CG17896	0.000384	eIF2beta	0.00814
66	boss	0.087	CG1907	0.027024	eIF3b	0.00033
67	brun	0.388	CG2233	1.38e-08	eIF3h	2.69e-06
68	Bsg	0.118	CG2767	0.000143	eIF3i	7.95e-08
69	bt	0.593	CG2852	0.017101	eIF4A	0.02929
70	bur	0.449	CG31098	0.003618	eIF4B	0.00065
71	BVR	0.422	CG31974	0.000318	Eno	0.00359
72	CaBP1	0.546	CG32017	0.000018	Epac	0.0169
73	CalpA	0.31	CG32225	0.000058	Eps-15	0.00132
74	CanA-14F	0.323	CG33307	0.01806	fax	0.00076
75	Cand1	0.065	CG34132	0.000566	Fib	0.04613
76	capt	0.077	CG3603	0.019649	Gale	0.0001
77	cass	0.253	CG3663	0.022864	Galphaq	0.0463
78	CCT1	0.173	CG42325	0.021558	Gbeta5	0.01775
79	CCT2	0.448	CG42361	0.053065	GCS2alpha	0.00085
80	CCT3	0.473	CG42540	0.009006	Gel	0.00167
81	CCT4	0.454	CG42683	0.038255	Ggamma30A	0.00276
82	CCT5	0.353	CG4408	0.005115	Gint3	0.01388
83	CCT6	0.266	CG4598	0.002253	Got1	0.00279
84	CCT7	0.057	CG4829	0.032724	Grx3	0.02555
85	CCT8	0.189	CG5023	0.028931	GstE12	0.0058
86	CD98hc	0.744	CG5254	0.010965	Gtpx	0.03422
87	Cdc37	0.871	CG5569	0.047127	Hn	4.07e-07
88	Cdc42	0.784	CG5958	0.046478	HnRNP-K	0.00226
89	Cds	0.243	CG6329	0.013093	how	0.00109
90	CG10126	0.123	CG6409	0.000262	Hsc70-4	0.00389
91	CG10132	0.133	CG7550	7.78e-07	Hsc70-5	0.00008
92	CG10135	0.113	CG7611	0.007024	Hsp110	0.00478
93	CG10550	0.56	CG8128	0.012276	Hsp23	0.00066
94	CG10737	0.529	CG8369	0.003323	Hsp26	0.00013
95	CG10863	0.109	CG8446-RE	0.004406	Hsp68	5.58e-07

96	CG1090	0.09	CG8475	0.035199	Hsp70Aa	0.00047
97	CG11089	0.259	CG8665	0.002129	Hsp83	0.00031
98	CG11134	0.203	CG8778	0.025579	HspB8	0.00616
99	CG11307	0.217	CG8993	0.000879	hts	0.02351
100	CG11625	0.633	CG9059	0.000062	Idgf3	0.03297
101	CG11883	0.673	CG9281	0.026922	Jhbp13	0.04482
102	CG11999	0.338	CG9331	0.043872	jub	0.00008
103	CG12123	0.103	CG9394	0.029772	KrT95D	0.02072
104	CG12237	0.08	CG9399	0.011606	MAPT	0.00027
105	CG12279	0.166	CG9436	0.011323	mdlc	0.00865
106	CG12562	0.291	CG9512	0.001818	Mp20	0.00002
107	CG13220	0.146	CG9813	0.009013	mRpL12	0.00464
108	CG13498	0.59	CG9928	0.006408	Mthfs	0.01738
109	CG13743	0.648	Chd64	0.012719	ND-30	0.05163
110	CG13928	0.542	chp	0.019045	ND-39	0.00075
111	CG14005	0.209	Cisd2	0.020632	ND-51	0.00881
112	CG14109	0.165	ClpP	0.021551	ND-75	0.03748
113	CG1416	0.239	CNBP	0.009728	ND-B14.5A	0.00283
114	CG14630	0.383	Cnx99A	0.000152	nudC	0.00267
115	CG14933	0.245	colt	0.014248	Nup93-2	0.0048
116	CG15293	0.642	Cont	0.047179	Nurf-38	0.00017
117	CG15602	0.407	COX5A	0.00029	Obp56d	0.04316
118	CG15747	0.246	COX7B	0.02873	Obp99c	0.01139
119	CG15772	0.427	Cpr47Ea	0.000951	Opa1	0.00037
120	CG1635	0.75	Cpr49Aa	0.000725	par-1	0.02218
121	CG1648	0.592	Cpr49Ab	0.008357	Pdhb	0.03727
122	CG16772	0.43	Cpr49Ae	0.033348	Pdxk	0.02941
123	CG16898	0.084	Cpr49Ag	0.000058	pont	0.01589
124	CG16935	0.283	Cpr62Bc	0.000029	Pp2A-29B	0.00317
125	CG17002	0.371	Cpr65Au	0.016402	PPO2	0.00273
126	CG17338	0.089	Cpr67B	0.000537	Prosap	0.01886
127	CG17816	0.924	CR14499	0.000449	Prx5	0.04243
128	CG17837	0.522	c-SPH35	5.28e-07	Psa	0.00012
129	CG17926	0.083	CT19169	0.00198	Pu	0.00373
130	CG1910	0.508	CT30783	0.001279	pzg	0.00005
131	CG1983	0.828	Cyp1	0.033616	Ref1	0.00147
132	CG2082	0.099	Cyt-b5-r	0.037132	Rop	0.03116
133	CG2269	0.136	Cyt-c1	0.002497	RpL12	0.01108
134	CG2915	0.572	Cyt-c-p	0.000624	RpL23	0.00001
135	CG3009	0.2	Dbct	0.004507	RpLP0	0.0001
136	CG30197	0.881	Dhrs4	0.001854	RpLP2	0.00001
137	CG31063	0.309	Dic1	0.000017	Rpn1	0.01068
138	CG31140	0.247	dj-1beta	0.039033	Rpn10	0.01352
139	CG31251	0.115	dlg1	0.009624	Rpn12	0.00227
140	CG31313	0.073	Dpp10	0.000069	Rpn13	1.59e-07
141	CG31548	0.08	e	0.011191	Rpn2	0.00134
142	CG3226	0.243	Eb1	0.047281	Rpn3	0.00008
143	CG33722	0.251	Echs1	0.012798	Rpn7	0.01672
144	CG34454	0.403	eIF3g1	0.016022	Rpn9	0.04114

145	CG3950	0.837	eIF3j	0.007891	RpS10b	0,00000
146	CG4038	0.212	EndoB	0.008079	RpS14b	0.00001
147	CG4115	0.358	Etfb	0.027648	RpS15	0.00028
148	CG4119	0.537	eys	0.046383	RpS21	0.01222
149	CG43346	0.473	Fas1	0.000235	RpS24	0.02091
150	CG4467	0.157	FASN1	0.001021	RpS3A	0.01184
151	CG45076	0.1	Fatp1	0.003377	RpS7	0.00152
152	CG4572	0.115	Fife	0.01646	Rrp6	0.00049
153	CG4588	0.783	Fkbp12	0.003692	Sdb	2.31e-07
154	CG4612	0.167	FOHSDR	0.031428	SdhA	0.00029
155	CG5364	0.157	frm	0.000427	se	3.32e-06
156	CG5504	0.954	fwe	0.044535	SerRS	0.03039
157	CG5618	0.324	Gabat	0.003676	sgg	0.00365
158	CG5641	0.229	Gad1	0.035934	Sirt1	0.00016
159	CG5946	0.135	gammaSnap1	0.042543	Ski6	0.00002
160	CG6005	0.757	Gapdh2	0.000515	slf	1.35e-09
161	CG6144	0.861	Gat	0.02218	Snapin	0.01115
162	CG6180	0.473	Gbeta13F	0.004169	sqd	0.00707
163	CG6195	0.803	GCR1	0.002642	sqh	0.00527
164	CG6330	0.092	Gdh	0.000524	Ssrp	3.04e-06
165	CG6638	0.871	Ggamma1	0.001683	su(r)	0.00003
166	CG6983	0.102	GH07925p	0.000028	Syn	0.0061
167	CG7139	0.247	glob1	0.010428	t	0.00012
168	CG7296	0.41	Glyp	0.001125	TER94	0.0005
169	CG7322	0.322	Grx5	0.008162	Tkt	0.00009
170	CG7409	0.143	GS	0.000086	Tpi	0.0001
171	CG7519	0.898	Gs1	0.000031	Trx-2	0.001
172	CG7632	0.725	Gs2	0.020466	Tsp	0.01558
173	CG7646	0.137	GstD1	0.000742	tsr	5.58e-07
174	CG7675	0.521	GstD2	0.002502	twin	0.01425
175	CG7692	0.182	GstD3	0.003916	veli	0.00014
176	CG7766	0.99	GstD4	0.000706	Vha26	0.01049
177	CG7920	0.914	GstD9	1.25e-06	Vha44	0.00594
178	CG8132	0.576	GstE1	0.003626	Vha55	0.00536
179	CG8187	0.116	GstE3	0.020392	Vha68-1	0.00836
180	CG8209	0.629	gw	0.021492	Vha68-2	0.00004
181	CG8607	0.271	Hasp	0.002882	yps	0.02961
182	CG8728	0.096	HDAC6	0.035481	Zasp66	0.0027
183	CG8768	0.059	Hex-C	0.000391		
184	CG8888	0.072	hgo	0.000023		
185	CG9132	0.154	HINT1	0.000114		
186	CG9150	0.407	His1	0.008993		
187	CG9231	0.899	His2B	0.044841		
188	CG9297	0.298	Hmu	0.011305		
189	CG9577	0.639	homer	0.000182		
190	CG9646	0.114	Hrb27C	0.050733		
191	CG9775	0.205	Hsdl2	0.0216		
192	ChAT	0.814	Hsp22	0.005361		
193	Chc	0.346	HtrA2	0.000109		

194	Chchd3	0.201	Hyccin	0.002249		
195	chic	0.155	IA-2	0.000034		
196	cindr	0.876	Idgf2	0.002548		
197	CkIIbeta	0.452	Idgf4	0.001191		
198	Clc	0.273	Idh	0.000025		
199	Clic	0.348	IM33	0.000678		
200	ClpX	0.603	Imp	0.003458		
201	Cmpk	0.448	inaF-B	0.013941		
202	Cndp2	0.749	jeb	0.000733		
203	comt	0.522	Jhbp14	0.029933		
204	cora	0.412	Jhedup	0.006526		
205	coro	0.748	Kap-alpha3	0.049421		
206	COX4	0.122	kcc	0.000038		
207	COX6B	0.557	l(2)k04810	0.00009		
208	COX7A	0.264	levy	0.048044		
209	Cp1	0.066	LManII	0.028078		
210	cpa	0.969	Lsp1beta	0.006956		
211	cpb	0.656	lva	0.019738		
212	cpo	0.793	mbf1	0.00228		
213	Cpr	0.124	Mcad	0.000502		
214	CPT2	0.061	Mdh2	0.005359		
215	cpx	0.22	Men	0.000246		
216	CRMP	0.363	Mhc	0.001129		
217	Csl4	0.571	Mitofilin	0.005351		
218	CSN3	0.082	Mlc2	0.050239		
219	CT9987	0.53	Mlc-c	0.015971		
220	CtBP	0.517	Mpcp2	0.000076		
221	ctp;Cdlc2	0.17	mt:ATPase8	0.00235		
222	CtsK1	0.379	mt:CoII	0.011521		
223	CtsK2	0.791	mtd	0.026251		
224	Cyp28d1	0.156	Mtpbeta	0.009668		
225	Cyp6d5	0.248	NaCP60E	0.005354		
226	Cyp9b2	0.79	Nagk	0.004367		
227	Cyp9f2	0.364	Nca	0.003291		
228	cype	0.11	ND-20	0.000012		
229	Cys	0.332	ND-B14.7	0.022652		
230	Cyt-b5	0.057	ND-B16.6	0.028562		
231	D1	0.54	ND-B17	0.01706		
232	D2hgdh	0.109	ND-MLRQ	0.032027		
233	DAAM	0.284	nec	0.014524		
234	Dap160	0.241	Nfs1	0.010468		
235	DCTN2-p50	0.128	ninaA	0.001069		
236	deltaCOP	0.706	ninaC	0.000614		
237	Dera	0.154	ninaE	0.007653		
238	Desat1	0.188	Nipsnap	0.000378		
239	Dhod	0.151	Npc2g	0.021305		
240	Dhpr	0.074	nrv2	0.011393		
241	Dlic	0.075	nrv3	0.000032		
242	DnaJ-1	0.395	Nt5b	0.024518		

243	dnc	0.16	NUCB1	0.020506		
244	dod	0.128	nwk	0.038582		
245	Dp1	0.239	Obp19d	0.014014		
246	Drat	0.541	Obp99a	0.000533		
247	Drp1	0.87	Ogdh	0.000648		
248	E3	0.352	Oscillin	0.026498		
249	Eaat1	0.599	p23	0.00005		
250	EbpIII	0.497	P32	0.022371		
251	eEF1gamma	0.13	P5cr	0.042238		
252	eEF2	0.283	P5cr-2	0.004553		
253	eEF5	0.311	P5CS	8.52e-08		
254	EG:103E12.2	0.265	PCB	8.50e-08		
255	EG:22E5.5	0.194	Pgd	0.004138		
256	EG:BACR7A4.14	0.886	Pgk	0.010298		
257	Egm	0.055	Pglym78	0.002173		
258	eIF1	0.167	Pgm1	0.00954		
259	eIF2gamma	0.977	Phb1	0.019401		
260	eIF3a	0.106	Phb2	0.014517		
261	eIF3c	0.381	pic	0.019514		
262	eIF3d1	0.171	Pisd	0.016448		
263	eIF3f1	0.512	Pkc98E	0.028675		
264	eIF3k	0.067	pkm	0.022788		
265	eIF3m	0.969	Pli	0.001126		
266	eIF4E1	0.486	PMCA	0.001903		
267	eIF4G1	0.158	pnut	0.007685		
268	EloB	0.303	porin	0.000594		
269	EMC8-9	0.097	Pp1alpha-96A	0.001707		
270	EndoA	0.091	PPO1	0.009517		
271	endos	0.753	primo-1	0.04367		
272	ERp44	0.273	Pripl4	0.000339		
273	ERp60	0.499	prom	0.022129		
274	Est-6	0.095	PyK	0.002574		
275	Esyt2	0.606	Rab32	0.005746		
276	Etf-QO	0.059	Rab39	0.011324		
277	fabp	0.376	Rack1	0.015161		
278	fbp	0.136	Rala	0.006626		
279	Fdh	0.346	Ran	0.001909		
280	FeCh	0.157	Ras85D	0.043035		
281	Fer1HCH	0.295	Rbcn-3A	0.001245		
282	Fer2LCH	0.072	rdgA	0.000873		
283	Fkbp59	0.331	Reg-2	5.5e-09		
284	flap	0.075	regucalcin	0.000944		
285	flr	0.111	RFeSP	0.026887		
286	flw	0.797	rogdi	0.000109		
287	FOHSDR	0.534	Rpi	1.34e-06		
288	fon	0.098	RpL14	0.038907		
289	for	0.194	RpL17	0.000572		
290	Frq1	0.12	RpL19	0.006898		

291	Fum1	0.104	RpL21	0.012014		
292	futsch	0.087	RpL26	0.026309		
293	Galk	0.749	RpL27	0.052731		
294	Galphao	0.189	RpL28	0.007353		
295	Galphas	0.404	RpL32	0.016855		
296	Galt	0.322	RpL6	0.048273		
297	gammaCOP	0.861	Rpt4	0.028894		
298	gammaTub23C	0.456	rtp	1.62e-06		
299	Gapdh1	0.067	ry	0.024746		
300	Gbeta76C	0.073	RyR	0.028656		
301	Gbp2	0.242	Sam-S	0.052574		
302	GC1	0.082	Sc2	0.00039		
303	Gclm	0.968	scf	0.004518		
304	GCS2beta	0.067	Scp1	0.000312		
305	Gdi	0.26	Scp2	0.020405		
306	gfzf	0.418	ScpX	0.004197		
307	GH26	0.588	Scsalpha1	0.027789		
308	Glo1	0.13	ScsbetaA	0.000387		
309	GLS	0.582	scu	0.005473		
310	GluProRS	0.118	SdhC	0.000337		
311	GlyRS	0.131	sea	0.000588		
312	Glys	0.404	sesB	0.050819		
313	Got2	0.638	Sfxn1-3	0.003871		
314	Gp93	0.309	Shab	0.000544		
315	Gpdh1	0.082	Shal	0.000155		
316	Gpo1	0.073	shv	0.042596		
317	Grip75	0.981	SkpA	0.015202		
318	Grip84	0.377	slgA	0.000467		
319	Grip91	0.356	Snap25	0.000955		
320	grsm	0.118	Sod1	0.000368		
321	Gss2	0.195	SpdS	0.000196		
322	GstE2	0.452	spidey	0.045835		
323	GstS1	0.611	Spn43Ab	0.000244		
324	GstT1	0.381	Sps1	0.039452		
325	GstZ2	0.133	Sqor	0.019938		
326	Gycalpha99B	0.063	Ssadh	1.14e-07		
327	Gyg	0.162	Stip1	0.027175		
328	Had1	0.348	Stoml2	0.00002		
329	Hao	0.437	sun	0.001668		
330	HDAC3	0.085	svr	0.000055		
331	Hel25E	0.175	sxe2	0.013336		
332	Hex-A	0.879	Syngr	0.00129		
333	Hexo2	0.757	Syp	0.011596		
334	hfp	0.349	Syt1	0.005576		
335	His2A	0.121	tfc	0.006977		
336	His3	0.47	TfIIB	0.00006		
337	His4	0.343	Tm1	0.026139		
338	HisT	0.116	tmod	0.007939		
339	hlk	0.056	TppII	0.039613		

340	hoe1	0.075	trus	0.00143		
341	Hou	0.133	Tsf1	2.98e-08		
342	Hpd	0.769	TwdlT	0.000667		
343	Hrb98DE	0.195	Ugt49B1	0.051138		
344	Hsc70-1	0.814	UK114	0.00838		
345	Hsc70-3	0.091	unc-13	0.014309		
346	Hsp27	0.296	UQCR-6.4	0.001077		
347	Hsp60A	0.21	UQCR-C1	0.01202		
348	Idgf1	0.367	UQCR-C2	0.021806		
349	Idgf6	0.118	UQCR-Q	0.010597		
350	Idh3a	0.493	Vap33	0.048998		
351	Idh3b	0.08	vib	0.030245		
352	Idh3g	0.198	wal	0.000027		
353	igl	0.201	wds	0.0013		
354	Ih	0.829	wupA	0.010918		
355	IleRS	0.099	yellow-f2	0.009705		
356	Ilk	0.396	yip2	0.010617		
357	inaC	0.07	Yp1	0.002557		
358	inaD	0.412	Yp2	0.004938		
359	Inos	0.057	Yp3	0.000031		
360	Inx2	0.107				
361	IPP	0.394				
362	isoQC	0.248				
363	Jabba	0.278				
364	jdp	0.117				
365	jef	0.558				
366	Jupiter	0.926				
367	Karybeta3	0.355				
368	Kdm5	0.646				
369	kdn	0.182				
370	Khc	0.133				
371	Khc-73	0.286				
372	Klc	0.685				
373	Kr-h2	0.119				
374	Ktl	0.238				
375	l(1)G0196	0.09				
376	l(1)G0320	0.32				
377	l(2)efl	0.259				
378	l(2)k09913	0.334				
379	l(3)04053	0.394				
380	L2HGDH	0.219				
381	Lam	0.212				
382	lap	0.262				
383	Lasp	0.105				
384	Ldh	0.393				
385	Ldsdh1	0.156				
386	Letm1	0.586				
387	lic	0.616				
388	lig	0.993				

389	LKRSDH	0.562				
390	Lmpt	0.191				
391	lost	0.463				
392	Lrp4	0.08				
393	Lsd-1	0.604				
394	Lsd-2	0.431				
395	mAcon1	0.066				
396	MagR	0.208				
397	MAPk-Ak2	0.831				
398	Mdh1	0.228				
399	me31B	1				
400	mEFTu1	0.652				
401	Men-b	0.351				
402	metro	0.177				
403	Mf	0.102				
404	mfas	0.256				
405	Mfe2	0.133				
406	Mgstl	0.054				
407	Mic26-27	0.137				
408	mip40	0.313				
409	Mkk4	0.219				
410	Mlp60A	0.738				
411	Mlp84B	0.698				
412	Mnr	0.204				
413	Mo25	0.067				
414	Moe	0.159				
415	mor	0.164				
416	Mpcp1	0.737				
417	Mpp6	0.104				
418	MRP	0.393				
419	mRpS18C	0.345				
420	MSBP	0.229				
421	Mtap	0.115				
422	Mtch	0.22				
423	Mtpalpha	0.426				
424	Mtr3	0.532				
425	mts	0.232				
426	mub	0.056				
427	muc	0.328				
428	Nacalpha	0.655				
429	Nap1	0.343				
430	Naprt	0.243				
431	Naxe	0.428				
432	ND-13A	0.36				
433	ND-15	0.185				
434	ND-18	0.519				
435	ND-19	0.068				
436	ND-23	0.753				
437	ND-24	0.784				

438	ND-42	0.283				
439	ND-49	0.263				
440	ND-ACP	0.289				
441	ND-B14	0.464				
442	ND-B17.2	0.767				
443	Ndf	0.439				
444	ND-PDSW	0.105				
445	Neb-cGP	0.145				
446	nebu	0.054				
447	NimB2	0.159				
448	NLaz	0.082				
449	Nlp	0.065				
450	nolo	0.076				
451	norpA	0.767				
452	Nplp2	0.484				
453	Nrg	0.486				
454	nrv1	0.607				
455	Nrx-IV	0.384				
456	Nsf2	0.121				
457	Nsun2	0.3				
458	nSyb	0.086				
459	Ntf-2	0.5				
460	Nup44A	0.621				
461	Obp44a	0.162				
462	Obp56e	0.745				
463	Obp99b	0.179				
464	ogre	0.909				
465	OtopLa	0.544				
466	ox	0.226				
467	p47	0.082				
468	P5CDh1	0.083				
469	pAbp	0.148				
470	Paf-AHalpha	0.339				
471	Paics	0.167				
472	Papss	0.304				
473	Past1	0.153				
474	path	0.326				
475	Pax	0.25				
476	Pcd	0.365				
477	pdgy	0.892				
478	Pdh	0.072				
479	Pdha	0.487				
480	Pdi	0.798				
481	Pdk	0.108				
482	Pef	0.847				
483	Pep	0.064				
484	pepCG10602	0.073				
485	Pfas	0.179				
486	Pfdn2	0.933				

487	Pfk	0.581				
488	Pfrx	0.955				
489	Pgi	0.077				
490	Pgm2a	0.866				
491	PIP4K	0.361				
492	PIP82	0.095				
493	Pis	0.217				
494	Pka-C1	0.61				
495	Pka-R1	0.909				
496	Pka-R2	0.579				
497	Pkc53E	0.173				
498	Plc21C	0.372				
499	POLDIP2	0.771				
500	Polr2l	0.817				
501	Pp1-13C	0.668				
502	Pp2B-14D	0.097				
503	Prat2	0.645				
504	Prm	0.139				
505	Prosalpha3	0.059				
506	Prp19	0.059				
507	Prps	0.422				
508	prtp	0.441				
509	Prx2	0.894				
510	Prx3	0.234				
511	Prx4	0.223				
512	Prx6b	0.968				
513	ps	0.601				
514	pug	0.235				
515	Pur-alpha	0.307				
516	pyd3	0.08				
517	QC	0.062				
518	QIL1	0.322				
519	qm	0.368				
520	Rab1	0.397				
521	Rab11	0.633				
522	Rab2	0.054				
523	Rab3	0.192				
524	Rab5	0.851				
525	Rab7	0.763				
526	Rac1	0.863				
527	Rae1	0.064				
528	Rap1	0.518				
529	Rap2l	0.175				
530	Rbcn-3B	0.062				
531	Rbp1-like;Rbp1	0.208				
532	rdgB	0.278				
533	rdgC	0.081				
534	rdhB	0.199				
535	retinin	0.081				

536	Rfk	0.917				
537	RH61753p	0.16				
538	Rho1	0.266				
539	ringer	0.222				
540	Roe1	0.778				
541	roh	0.239				
542	RpL10Ab	0.431				
543	RpL11	0.294				
544	RpL13	0.139				
545	RpL15	0.413				
546	RpL18	0.233				
547	RpL18A	0.438				
548	RpL22	0.449				
549	RpL23A	0.139				
550	RpL24	0.322				
551	RpL27A	0.057				
552	RpL3	0.252				
553	RpL30	0.99				
554	RpL31	0.63				
555	RpL34a;RpL34b	0.311				
556	RpL36	0.386				
557	RpL38	0.658				
558	RpL4	0.171				
559	RpL5	0.25				
560	RpL7	0.885				
561	RpL7A	0.079				
562	RpL8	0.43				
563	RpL9	0.611				
564	RpLP1	0.335				
565	Rpn11	0.206				
566	Rpn5	0.781				
567	Rpn6	0.686				
568	Rpn8	0.912				
569	RpS11	0.222				
570	RpS12	0.1				
571	RpS13	0.725				
572	RpS15Aa;RpS15Ab	0.542				
573	RpS16	0.174				
574	RpS17	0.215				
575	RpS18	0.715				
576	RpS19a	0.303				
577	RpS2	0.447				
578	RpS20	0.465				
579	RpS23	0.101				
580	RpS25	0.645				
581	RpS26	0.085				
582	RpS27	0.491				
583	RpS27A	0.77				
584	RpS28b	0.152				

585	RpS3	0.512				
586	RpS30	0.27				
587	RpS4	0.513				
588	RpS5a	0.307				
589	RpS6	0.449				
590	RpS8	0.083				
591	RpS9	0.683				
592	Rpt1	0.189				
593	Rpt2	0.717				
594	Rpt3	0.895				
595	Rpt5	0.197				
596	Rpt6;Rpt6R	0.256				
597	Rrp4	0.117				
598	Rrp40	0.429				
599	Rrp42	0.443				
600	Rrp45	0.313				
601	Rrp46	0.377				
602	Rrp47	0.961				
603	Rtnl1	0.208				
604	rush	0.779				
605	Sac1	0.068				
606	Sap47	0.587				
607	Sap-r	0.09				
608	Sar1	0.643				
609	SCaMC	0.405				
610	Sccpdh1	0.753				
611	ScsbetaG	0.713				
612	SdhB	0.184				
613	sds22	0.382				
614	Sec13	0.118				
615	Sec23	0.634				
616	Sec61alpha	0.732				
617	Septin2	0.242				
618	SERCA	0.252				
619	SF2	0.913				
620	Sfxn2	0.247				
621	sgll	0.669				
622	SH3PX1	0.234				
623	shi	0.102				
624	Shmt	0.136				
625	shrb	0.446				
626	sls	0.089				
627	SmD2	0.162				
628	SmD3	0.606				
629	SmE	0.412				
630	smg	0.061				
631	smid	0.988				
632	Smr	0.983				
633	SmydA-9	0.577				

634	sn	0.749				
635	Snap29	0.368				
636	Sod2	0.371				
637	Sodh1	0.33				
638	SP170	0.684				
639	SP99	0.964				
640	Sply	0.485				
641	Spn42Da	0.057				
642	Spn77Bb	0.097				
643	Srlp	0.101				
644	Srp19	0.977				
645	Srp54	0.456				
646	SsRbeta	0.078				
647	sta	0.596				
648	Stim	0.371				
649	stnB	0.231				
650	Strn-Mlck	0.506				
651	Su(var)205	0.107				
652	Synd	0.131				
653	Syt7	0.68				
654	Syx1A	0.748				
655	T3dh	0.089				
656	Taldo	0.063				
657	Tapdelta	0.329				
658	Tat	0.19				
659	tau	0.167				
660	Tctp	0.137				
661	Tep4	0.115				
662	tex	0.637				
663	tho2	0.389				
664	thoc6	0.12				
665	ThrRS	0.073				
666	Tim8	0.177				
667	Tm2	0.338				
668	Tmlh	0.212				
669	Tom70	0.639				
670	TpnC25D	0.164				
671	Tppl	0.451				
672	Tps1	0.278				
673	tral	0.546				
674	Treh	0.064				
675	Trhn	0.292				
676	trol	0.746				
677	trp	0.678				
678	Trs20	0.248				
679	Trs23	0.551				
680	Trxr1	0.152				
681	TSG101	0.767				
682	tws	0.419				

683	Txl	0.284				
684	tzn	0.966				
685	Uba1	0.24				
686	Uch	0.617				
687	Uch-L5	0.814				
688	Ufd1	0.142				
689	UGP	0.069				
690	unc-104	0.096				
691	Unc-115b	0.647				
692	Unc-89	0.596				
693	unk	0.552				
694	up	0.38				
695	UQCR-14	0.27				
696	Usp14	0.837				
697	Usp7	0.163				
698	VAChT	0.712				
699	Vago	0.093				
700	vari	0.625				
701	Vha100-1	0.259				
702	Vha13	0.943				
703	Vha14-1	0.23				
704	Vha36-1	0.731				
705	VhaAC39-1	0.106				
706	VhaSFD	0.59				
707	vir-1	0.173				
708	Vps13	0.212				
709	Vps28	0.618				
710	Vps35	0.074				
711	Vps4	0.145				
712	WASp	0.141				
713	Wdfy2	0.09				
714	Wdr37	0.229				
715	yellow-c	0.312				
716	Zasp52	0.548				
717	Zw	0.205				

Table S1. This table lists the complete set of 1,258 high-confidence interactors, identified in FLAG-2N4R Tau co-immunoprecipitations of adult *Drosophila* heads collected at 4, 8, and 12 days of induction and quantified by label-free LC-MS/MS. For each protein, we report the gene symbol, the multifactor ANOVA p value, and the temporal class assigned in the main text (Stable [grey], Early-enriched [peak at day 4; blue], or Late-enriched [peak at day 12; red]). Proteins with significant time-dependent changes ($p < 0.05$) correspond to those visualized in Figure 1B.

Table S2. GO Cellular Component enrichment.

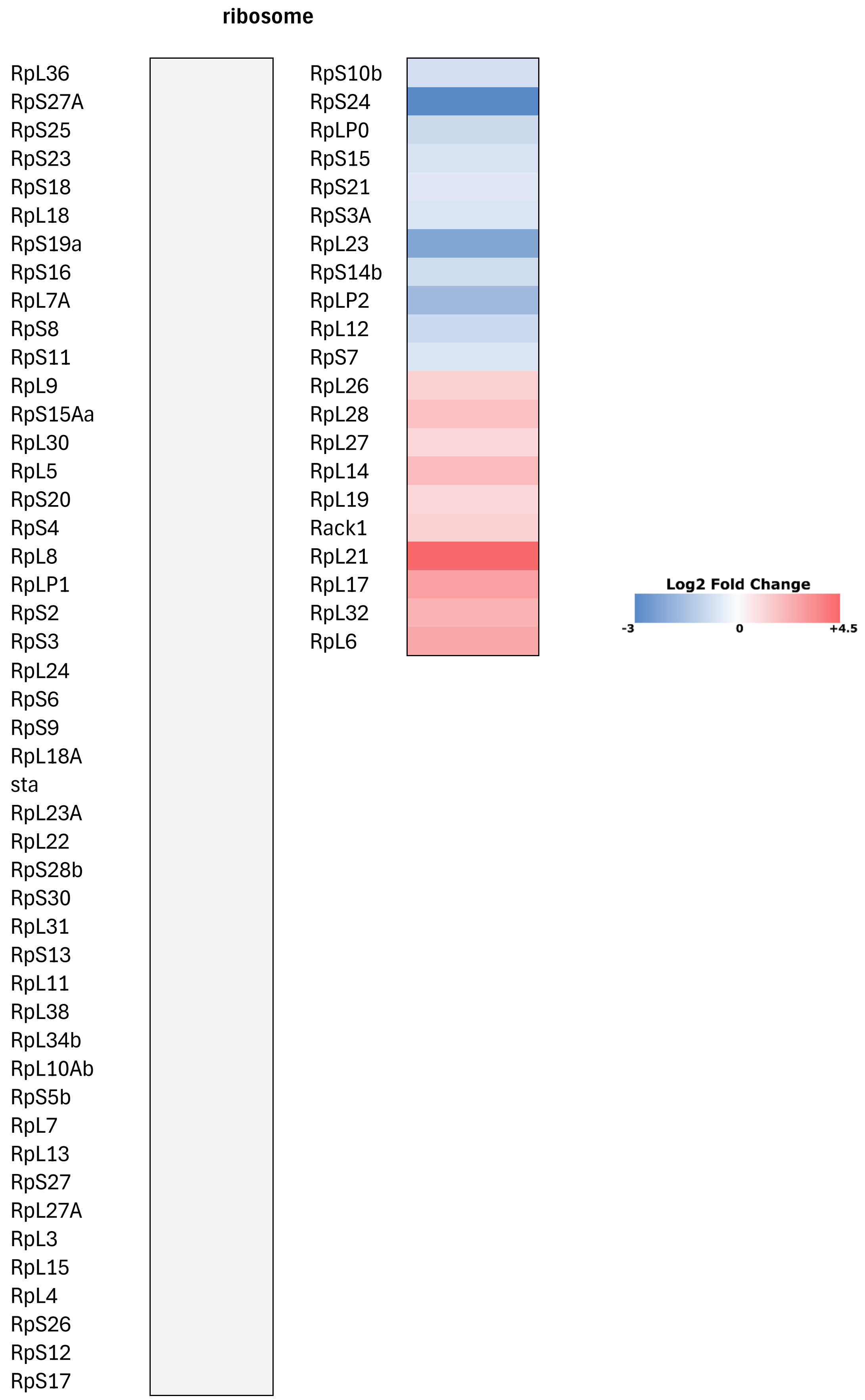

Table S2. Heat maps revealing the fold change in levels for the proteins of each cellular component GO-term in Figure 1C. The temporal class is the same as assigned in the main text (Stable [grey], Early-enriched [peak at day 4; blue], or Late-enriched [peak at day 12; red]).

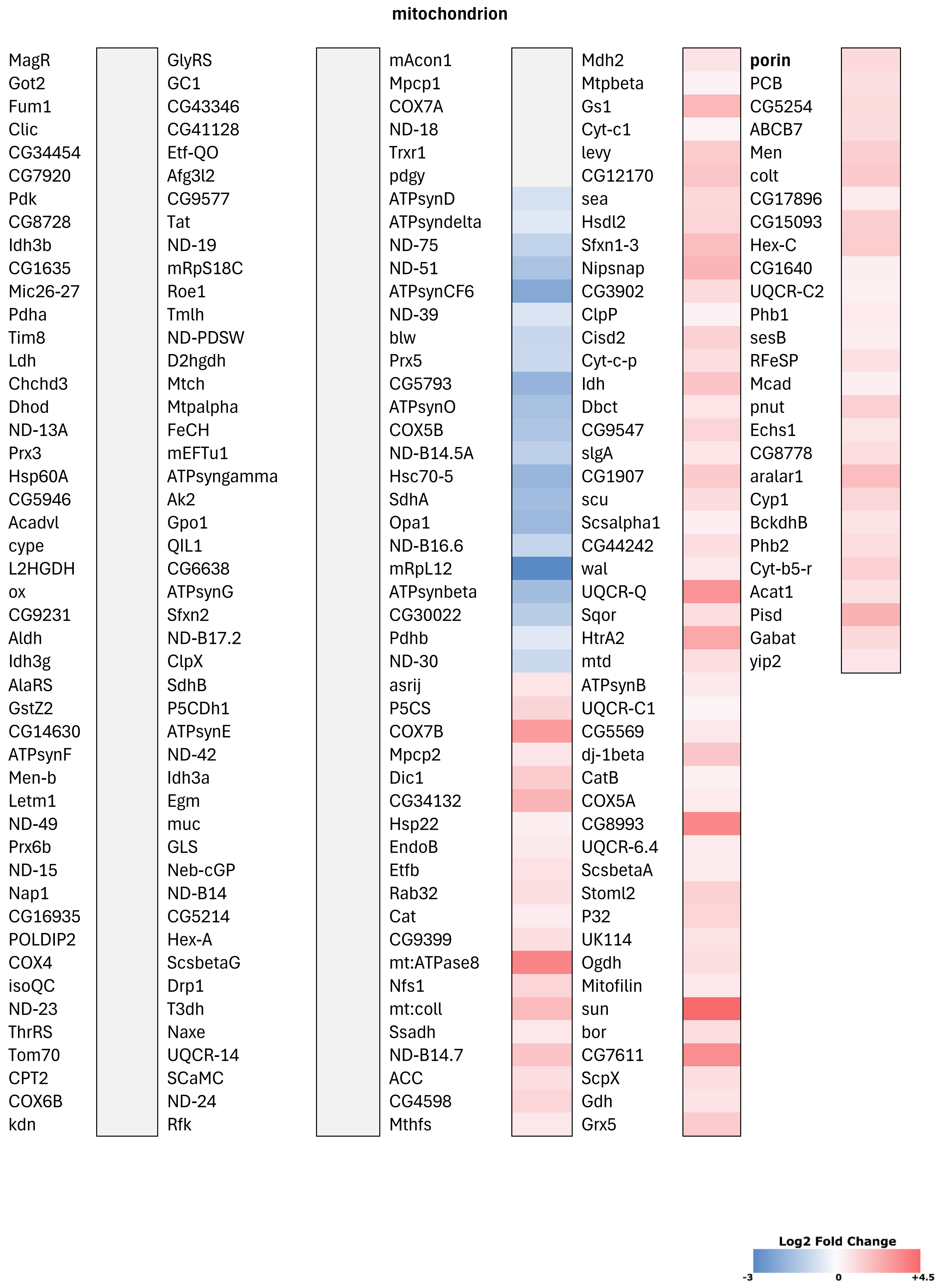
mitochondrion
MagR
Got2
Fum1
Clic
CG34454
CG7920
Pdk
CG8728
Idh3b
CG1635
Mic26-27
Pdha
Tim8
Ldh
Chchd3
Dhod
ND-13A
Prx3
Hsp60A
CG5946
Acadvl
cype
L2HGDH
ox
CG9231
Aldh
Idh3g
AlaRS
GstZ2
CG14630
ATPsynF
Men-b
Letm1
ND-49
Prx6b
ND-15
Nap1
CG16935
POLDIP2
COX4
isoQC
ND-23
ThrRS
Tom70
CPT2
COX6B
kdn
GlyRS
GC1
CG43346
CG41128
Etf-QO
Afg3l2
CG9577
Tat
ND-19
mRpS18C
Roe1
Tmlh
ND-PDSW
D2hgdh
Mtch
Mtpalpha
FeCH
mEFTu1
ATPsyngamma
Ak2
Gpo1
QIL1
CG6638
ATPsynG
Sfxn2
ND-B17.2
ClpX
SdhB
P5CDh1
ATPsynE
ND-42
Idh3a
Egm
muc
GLS
Neb-cGP
ND-B14
CG5214
Hex-A
ScsbetaG
Drp1
T3dh
Naxe
UQCR-14
SCaMC
ND-24
Rfk
mAcon1
Mpcp1
COX7A
ND-18
Trxr1
pdgy
ATPsynD
ATPsyndelta
ND-75
ND-51
ATPsynCF6
ND-39
blw
Prx5
CG5793
ATPsynO
COX5B
ND-B14.5A
Hsc70-5
SdhA
Opa1
ND-B16.6
mRpL12
ATPsynbeta
CG30022
Pdhb
ND-30
asrij
P5CS
COX7B
Mpcp2
Dic1
CG34132
Hsp22
EndoB
Etfb
Rab32
Cat
CG9399
mt:ATPase8
Nfs1
mt:coII
Ssadh
ND-B14.7
ACC
CG4598
Mthfs
Mdh2
Mtpbeta
Gs1
Cyt-c1
levy
CG12170
sea
Hsdl2
Sfxn1-3
Nipsnap
CG3902
ClpP
Cisd2
Cyt-c-p
Idh
Dbct
CG9547
slgA
CG1907
scu
Scsalpha1
CG44242
wal
UQCR-Q
Sqor
HtrA2
mtd
ATPsynB
UQCR-C1
CG5569
dj-1beta
CatB
COX5A
CG8993
UQCR-6.4
ScsbetaA
Stoml2
P32
UK114
Ogdh
Mitofilin
sun
bor
CG7611
ScpX
Gdh
Grx5
porin
PCB
CG5254
ABCB7
Men
colt
CG17896
CG15093
Hex-C
CG1640
UQCR-C2
Phb1
sesB
RFeSP
Mcad
pnut
Echs1
CG8778
aralar1
Cyp1
BckdhB
Phb2
Cyt-b5-r
Acat1
Pisd
Gabat
yip2
Log2 Fold Change
-3
0
+4.5

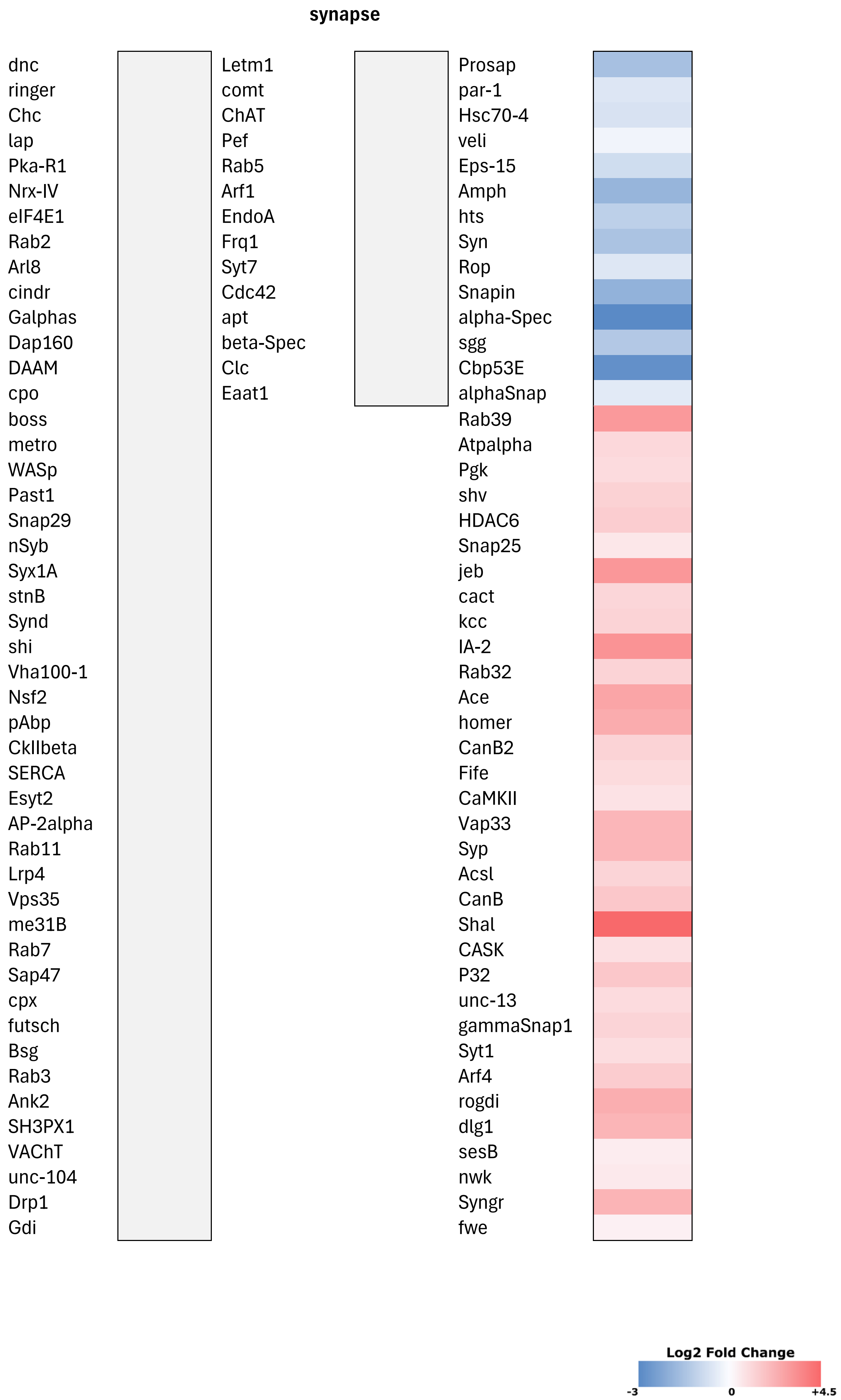
synapse
dnc
ringer
Chc
lap
Pka-R1
Nrx-IV
eIF4E1
Rab2
Arl8
cindr
Galphas
Dap160
DAAM
cpo
boss
metro
WASp
Past1
Snap29
nSyb
Syx1A
stnB
Synd
shi
Vha100-1
Nsf2
pAbp
CkIIbeta
SERCA
Esyt2
AP-2alpha
Rab11
Lrp4
Vps35
me31B
Rab7
Sap47
cpx
futsch
Bsg
Rab3
Ank2
SH3PX1
VAChT
unc-104
Drp1
Gdi
Letm1
comt
ChAT
Pef
Rab5
Arf1
EndoA
Frq1
Syt7
Cdc42
apt
beta-Spec
Clc
Eaat1
Prosap
par-1
Hsc70-4
veli
Eps-15
Amph
hts
Syn
Rop
Snapin
alpha-Spec
sgg
Cbp53E
alphaSnap
Rab39
Atpalpha
Pgk
shv
HDAC6
Snap25
jeb
cact
kcc
IA-2
Rab32
Ace
homer
CanB2
Fife
CaMKII
Vap33
Syp
Acsl
CanB
Shal
CASK
P32
unc-13
gammaSnap1
Syt1
Arf4
rogdi
dlg1
sesB
nwk
Syngr
fwe
Log2 Fold Change
-3
0
+4.5

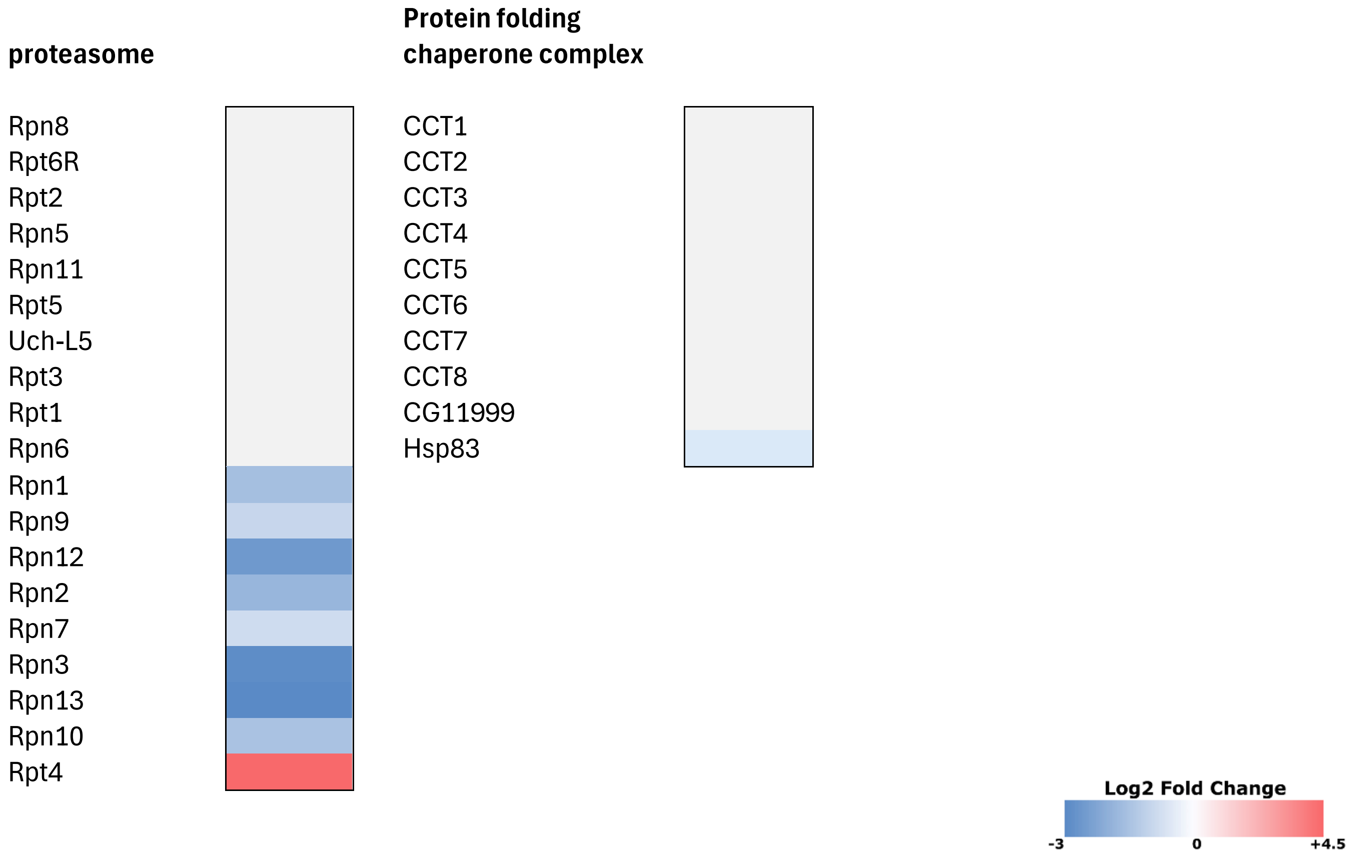
proteasome
Rpn8
Rpt6R
Rpt2
Rpn5
Rpn11
Rpt5
Uch-L5
Rpt3
Rpt1
Rpn6
Rpn1
Rpn9
Rpn12
Rpn2
Rpn7
Rpn3
Rpn13
Rpn10
Rpt4
Protein folding chaperone complex
CCT1
CCT2
CCT3
CCT4
CCT5
CCT6
CCT7
CCT8
CG11999
Hsp83
Log2 Fold Change
-3
0
+4.5

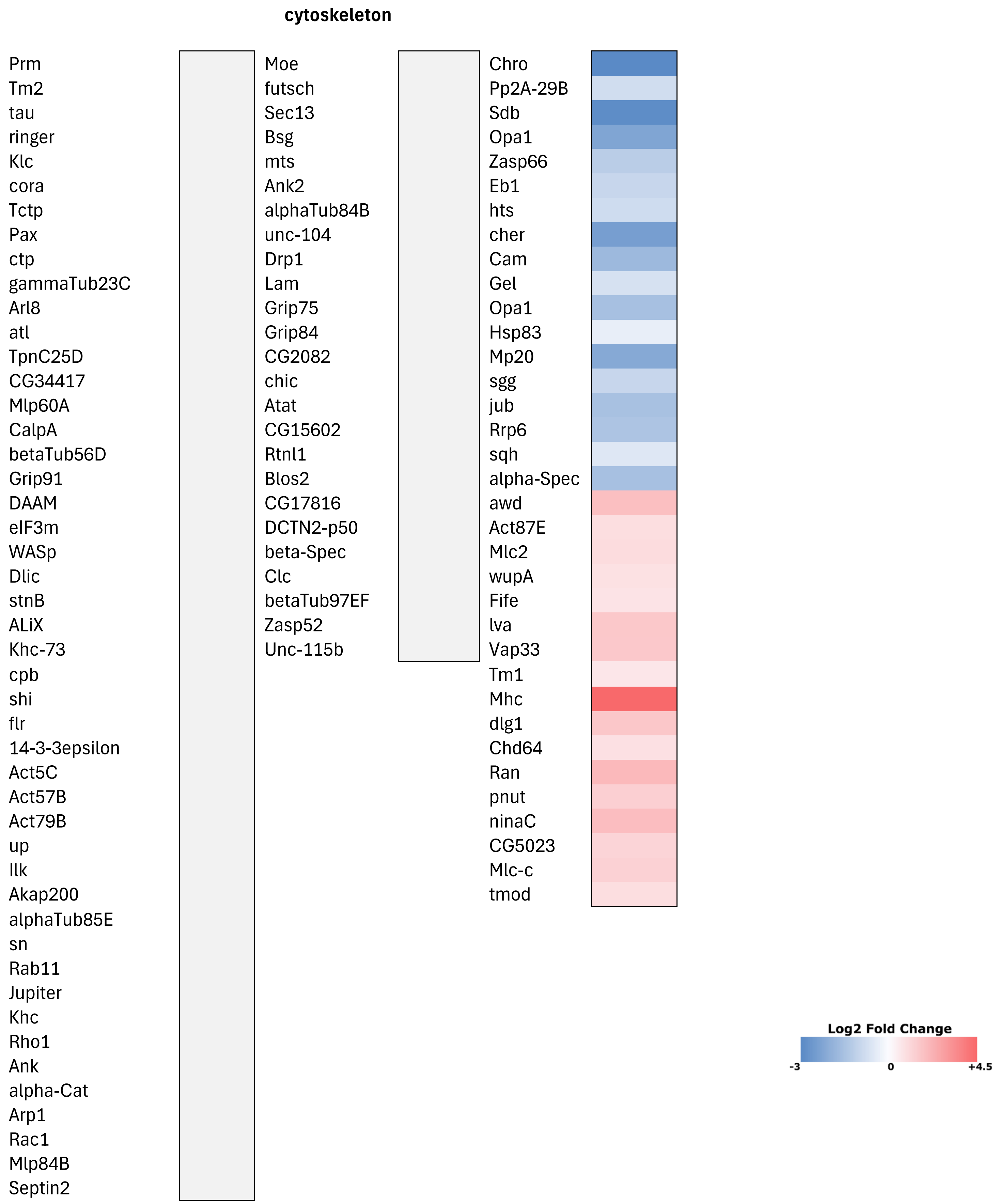

Table S2. Heat maps revealing the fold change in levels for the proteins of each cellular component GO-term in Figure 1C. The temporal class is the same as assigned in the main text (Stable [grey], Early-enriched [peak at day 4; blue], or Late-enriched [peak at day 12; red]).

Table S3

Comparisons to control Elav^{C155}-Gal4> w^{1118} and Elav^{C155}-Gal4>0N4R using LSM

FIGURE 4			
**Genotype**	**Mean ± SEM**	**F-Ratio**	**p**
**Figure 4D**	**ANOVA $F_{(3,38)}$= 32.4639,**	**p=3,2301e-10**	
Elav^{C155}; G80^{ts}-Gal4 > w^{1118}	51,3722± 1,807		
Elav^{C155}; G80^{ts}-Gal4 > Porin^{RNAi}	23,1931± 2,830	57,546	6,806e-9
Elav^{C155}; G80^{ts}-Gal4 >0N4R	27,9535± 3,043	42,155	1,742e-7
Elav^{C155}; G80^{ts}-Gal4 >0N4R; Porin^{RNAi}	18,097 ± 3,386	74,890	3,22e-10
Elav^{C155}; G80^{ts}-Gal4 >0N4R	27,9535± 3,043		
Elav^{C155}; G80^{ts}-Gal4 > Porin^{RNAi}	23,1931± 2,830	1,5126	0,226944
Elav^{C155}; G80^{ts}-Gal4 >0N4R; Porin^{RNAi}	18,097 ± 3,386	6,0834	0,018687

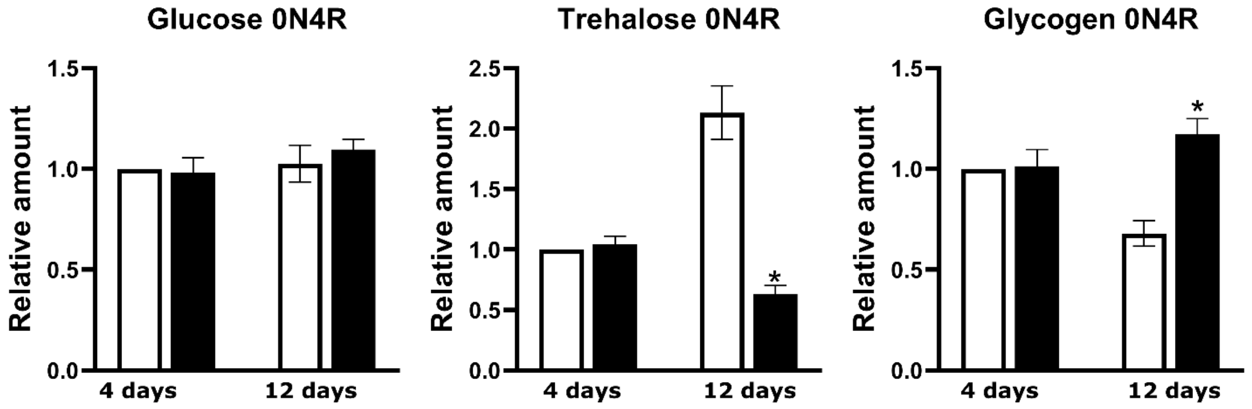

Figure S1. Brain trehalose, glucose and glycogen in 0N4R Tau-expressing flies at 4 d and 12 d mirror the 2N4R results (Figure 2D–F): Glucose levels in head homogenates at 4 and 12 days post-induction (mean ± SEM; n = 6) are unchanged (p>0.05). Trehalose fails to increase in Tau-expressing flies at 12 d (mean ± SEM; n = 5, * p= 0.0002 versus ctrl 12d). Control flies at 4d of induction have lower trehalose levels than controls at 12d (mean ± SEM; n = 5, p=0.0009). Glycogen levels demonstrate increased glycogen accumulation in Tau-expressing flies at day 12 (mean ± SEM; n = 6, * p = 0.0006 versus ctrl 12d). Control flies at 12d of induction have lower glycogen levels than those at 4d (mean ± SEM; n = 6, p=0.0005). Levels are normalized to control 4d. Data were analyzed using standard parametric statistics, with Student's t-tests performed for each comparison.

Figure 3A

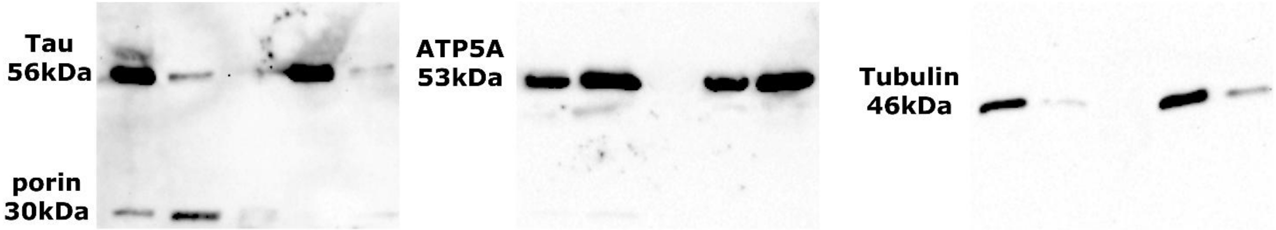

Figure 3B

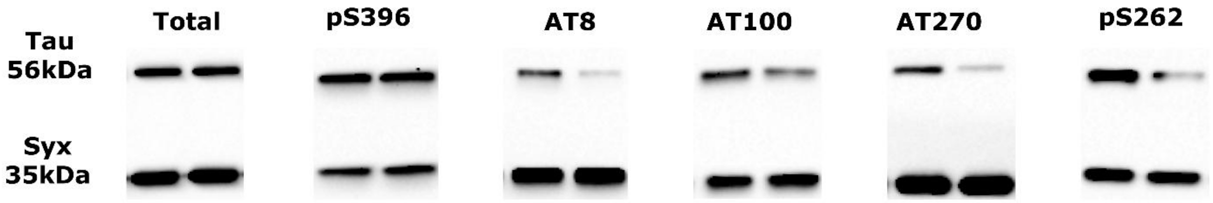

Figure 3C

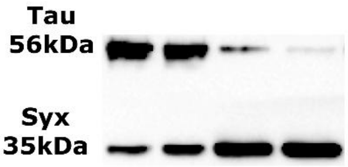

Figure 3D

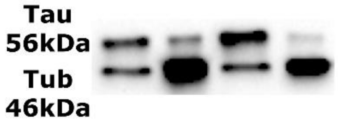

Figure S2. Uncropped western blot images corresponding to those presented in Figure 3 are shown. Molecular weights of the detected proteins are indicated alongside the blots.

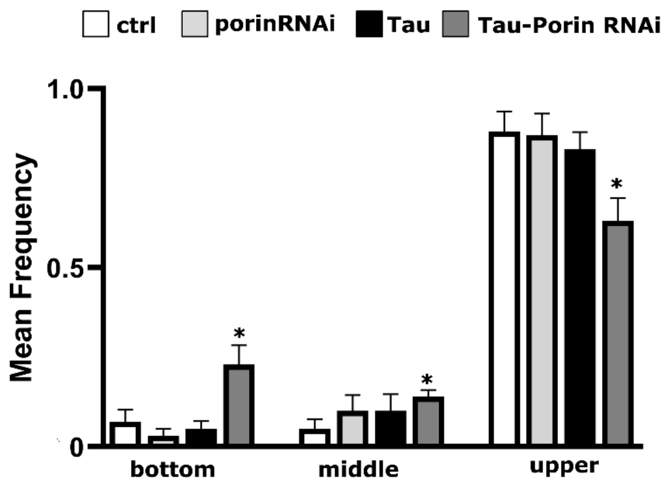

Figure S3. Short-vial negative geotaxis under a mild challenge is intact in single transgenics and only Tau + Porin RNAi shows a deficit. Adult-specific expression was induced under elavC155-Gal4; tub-Gal80^{ts} at 30 °C for 12 days. Flies were tapped to the bottom of a short vial subdivided into three 2-cm zones, and positions were scored 9 s later. Zone frequency (fraction of flies in each zone) is shown for control driver heterozygotes, Tau alone, Porin RNAi alone, and Tau + Porin RNAi double transgenics. Tau-only and Porin-only groups did not differ from controls across zones ($p > 0.05$), whereas the double transgenic was less likely to reach the upper zone ($p = 0.0191$) and more likely to occupy the lower ($p = 0.0361$) and middle ($p = 0.0265$) zones. Thus, baseline locomotion under a mild challenge is intact in Tau-only and Porin RNAi-only cohorts at 12 days, indicating that the learning impairments in these groups are not attributable to generalized motor dysfunction. Bars indicate mean ± SEM; p values from one-way ANOVA with Dunnett's multiple comparisons versus the control.
